# A single-cell atlas of adult *Drosophila* ovary identifies transcriptional programs and somatic cell lineage regulating oogenesis

**DOI:** 10.1371/journal.pbio.3000538

**Published:** 2020-04-27

**Authors:** Allison Jevitt, Deeptiman Chatterjee, Gengqiang Xie, Xian-Feng Wang, Taylor Otwell, Yi-Chun Huang, Wu-Min Deng

**Affiliations:** 1 Department of Biological Science, Florida State University, Tallahassee, Florida, United States of America; 2 Department of Biochemistry and Molecular Biology, Tulane University School of Medicine, New Orleans, Louisiana, United States of America; Carnegie Institute of Washington, UNITED STATES

## Abstract

Oogenesis is a complex developmental process that involves spatiotemporally regulated coordination between the germline and supporting, somatic cell populations. This process has been modeled extensively using the *Drosophila* ovary. Although different ovarian cell types have been identified through traditional means, the large-scale expression profiles underlying each cell type remain unknown. Using single-cell RNA sequencing technology, we have built a transcriptomic data set for the adult *Drosophila* ovary and connected tissues. Using this data set, we identified the transcriptional trajectory of the entire follicle-cell population over the course of their development from stem cells to the oogenesis-to-ovulation transition. We further identify expression patterns during essential developmental events that take place in somatic and germline cell types such as differentiation, cell-cycle switching, migration, symmetry breaking, nurse-cell engulfment, egg-shell formation, and corpus luteum signaling. Extensive experimental validation of unique expression patterns in both ovarian and nearby, nonovarian cells also led to the identification of many new cell type–and stage-specific markers. The inclusion of several nearby tissue types in this data set also led to our identification of functional convergence in expression between distantly related cell types such as the immune-related genes that were similarly expressed in immune cells (hemocytes) and ovarian somatic cells (stretched cells) during their brief phagocytic role in nurse-cell engulfment. Taken together, these findings provide new insight into the temporal regulation of genes in a cell-type specific manner during oogenesis and begin to reveal the relatedness in expression between cell and tissues types.

## Abstract 2

A single-cell RNA sequencing analysis of the Drosophila ovary identifies novel cell-type-specific signatures underlying the essential processes of oogenesis, including differentiation, cell cycle switching, morphogenesis, migration, symmetry breaking, phagocytosis, eggshell formation, oogenesis-to-ovulation shift, and corpus luteum formation.

## Introduction

The adult *Drosophila* ovary is a versatile model for the study of cell and developmental biology. Using the powerful genetic tools available in *Drosophila*, countless studies of oogenesis have provided mechanistic insight into broader biological topics such as stem cell niche regulation [1–6], cell differentiation [7, 8], cell cycle and size control [9, 10], epithelial morphogenesis [11–13], cell migration [14, 15], tissue repair and homeostasis [16, 17], etc. The success of this system as a developmental model is also due to the anatomy of *Drosophila* ovaries. As described below, many rounds of oogenesis occur simultaneously in each ovary, providing substantial replication of cell types. Temporal information for each cell type can also be collected from an individual fly because egg chambers physically progress in the ovary from anterior to posterior in a queue throughout developmental time. These experimental advantages make the ovary ideally suited for single-cell RNA sequencing (scRNA-seq) compared to other tissue types. Because ovaries are easily dissected, many ovaries can be pooled together in a single sample further increasing the robust biological replication of all cell types and developmental time points within one library.

A female fly has a pair of ovaries that are connected to the oviduct and held together by muscles known as the peritoneal sheath. Each ovary is made up of developmental units called ovarioles, which are individually sheathed within the musculature known as the epithelial sheath. Oogenesis occurs simultaneously within each of the 16 to 18 ovarioles, starting from stem cells at the anterior tip to the fully developed eggs at the posterior end. Throughout oogenesis, the developing egg is supported by the germline-derived nurse cells and the somatic follicular epithelium (made up of follicle cells). Together, the germline and follicle cells form individual units called egg chambers. Egg chamber development is subdivided into early (1–6), middle (7–10A), and late (10B–14) stages based on mitotic, endocycle, and gene amplification cell-cycle programs of the follicle cells, respectively [18]. During ovulation, mature eggs break free from the epithelium and pass into the uterus through the oviduct. The epithelial layer remains in the ovary, forming a structure similar to one found in mammals, known as the corpus luteum [19, 20].

To better understand how oogenesis is regulated at the cellular level, we performed scRNA-seq on these ovarian cell types and uncovered novel gene expression patterns throughout oogenesis. With a special focus on the follicle-cell trajectory, we also described the major transcriptomic programs underlying the early, middle, and late stages of oogenesis. We also identified the large-scale transcriptional shift in late-staged follicle cells (termed precorpus luteum cells) from egg-shell–related genes to ovulation-related genes, which occurs during oogenesis-to-ovulation transition.

## Materials and methods

### Experimental model

#### Fly lines used for ScRNA-seq

All fly stocks and crosses were maintained at room temperature (23 °C) and fed a yeast-based medium. To construct the scRNA-seq data set, ***w***^−^ flies (BL#3605) were used, a common genetic background used in many studies [21].

#### Fly lines used in experimental validation of cluster markers

We used a variety of publicly available lines from Bloomington Stock Center to experimentally validate expression patterns of select genes from the scRNA-seq data set. These lines fall into 2 categories: those with fluorescently tagged proteins under the control of a native promoter (either MiMIC-based RMCE [22] or protein trap [23]) and those expressing T2A-Gal4 (carrying either CRISPR-mediated insertions of T2A-Gal4 [24] or RMCE (recombinase-mediated cassette exchange)-mediated swap-ins of T2A-Gal4 [25]) driving UAS-GFP (BL#4775) or UAS-RFP (BL#31417) as a marker.

The GFP-tagged lines used in this study are Atf3:GFP (BL#42263), Ilp8:GFP (BL#33079), Past1:GFP (BL#51521), Glut4EF:GFP (BL#60555), abd-A:GFP (BL#68187), Chrac-16:GFP (BL#56160), shep:GFP (BL#61769), AdenoK:GFP (BL#56160), Fkbp1:GFP (BL#66358), mub:GFP (BL#51574), mnb:GFP (BL#66769), Gp210:GFP (BL#61651), Fpps:GFP (BL#51527), HmgD:GFP (BL#55827), sli:GFP (BL#64472), Nrx-IV:GFP (BL#50798), CG14207:GFP (BL#60226), D1:GFP (BL#66454), jumu:GFP (BL#59764), hdc:GFP (BL#59762), sm:GFP (BL#59815), Men:GFP (BL#61754), Sap-r:GFP (BL#63201), GILT1:GFP (BL#51543), Cp1:GFP (BL#51555). The T2A-Gal4 lines used in this study are Ance-Gal4 (BL#76676), FER-Gal4 (BL#67448), wb-Gal4 (BL#76189), stx-Gal4 (BL#77769), vir-1-Gal4 (BL#65650).

We also used Diap1:GFP, a kind gift from Jin Jiang Lab [26].

### Immunofluorescence and imaging

Ovaries and associated tissue were dissected in Phosphate-Buffered Saline (PBS), fixed for 15 minutes in 4% formaldehyde, washed 3 times in Phosphate-Buffered Saline and Tween 20 (PBT), and then stained with DAPI (Invitrogen, 1:1,000) to label nuclei. Samples were then mounted on slides in an 80% glycerol mounting solution. All images were captured using the Zeiss LSM 800 confocal microscope and associated Zeiss microscope software (ZEN blue).

### ScRNA-seq sample preparation

#### Dissociation and filtration of single cells

As described above, each ovary contains 16 to 18 replicates of oogenesis. However, to maximize sampling genetic diversity between individuals and adequately capturing rarer cell types, we dissected 100 ovaries from 50 adult flies. It is technically challenging to separate the ovaries from surrounding and interconnected tissues (i.e., fat body, muscle sheath, hemocytes, and oviduct) without damaging the ovarian cells. Thus, in order to minimize damage or death to ovarian cell types of interest, we elected to include these surrounding cell types in our analysis.

Female flies were selected on the day of eclosion and maintained at 25 °C with access to males and yeast supplement for 3 days (a common experimental condition in many studies). Flies were then dissected in complete medium (Grace's Insect Basal Medium supplemented with 15% fetal bovine serum). To prevent cell clumping, ovaries were transferred to a tube containing 300 μL Earle's Balanced Salt Solution (EBSS) (no calcium, magnesium, and phenol red) and gently washed for 2 minutes. The EBSS was then removed, and the tissue was dissociated in 100 μL Papain (50 U/mL in EBSS and previously heat activated in 37 °C for 15 minutes) for 30 minutes. The suspension was mechanically dissociated every 3 minutes by gentle pipetting up and down. To quench the digestion, 500 μL complete medium was added to dissociated cells. The suspension was then passed through a 40 μL sterile cell strainer and centrifuged for 10 minutes at 700 RCF to remove large eggs with intact egg shell which cannot be dissociated and debris. This also filtered out larger germline cells that increase dramatically in size around stage 9 [27]. Supernatant was removed and single cells were resuspended in 100 μL. Cell viability was assayed using Trypan Blue and estimates of cell concentration were made using a hemocytometer. Cells were then further diluted to an approximate, final concentration of 2,000 cells/μL according to 10X Genomics recommendations. Two technical replicates were generated in this way and sequenced separately.

#### 10X Genomics library preparation

Single-cell libraries were prepared for both technical replicates using the Single Cell 3' Library & Gel Bead Kit v2 and Chip Kit according to the recommended 10X Genomics protocol. Single-cell suspension was loaded onto the Chromium Controller (10X Genomics). Library quantification assays and quality check analysis was performed using the 2100 Bioanalyzer instrument (Agilent Technologies). The library samples were then diluted to a 10 nM concentration and loaded onto 2 lanes of the NovaSeq 6000 (Illumina) instrument flow cell for a 100-cycle sequencing run. A total of 429,855,892 reads were obtained with 28,995 mean reads per cell for replicate 1. Replicate 2 yielded 202,410,944 reads with 92,340 mean reads per cell (S1 Fig). We have only used the data set with greater sequencing depth and a greater number of cells (replicate 1) for all downstream analyses, while using replicate 2 to validate clusters and remove potential batch effects. Replicate 2 was not considered for downstream analyses for marker identification and pseudotemporal alignment alongside replicate 1, in order to prevent signal dropouts (because of a lack of comparable sequencing depth) from affecting marker enrichment in replicate 1.

### Quantification and statistical analysis

#### Preprocessing chromium scRNA-seq output

We processed the raw sequencing reads from each of the 10X Genomics Chromium single-cell 3' RNA-seq libraries using Cell Ranger (version 3.0.0), the recommended analysis pipeline from the Chromium single-cell gene expression software suite. The reference index for Cell Ranger was built using the *Drosophila melanogaster* Release 6 reference genome assembly [28] made available on the Ensembl genome database. The cellranger count pipeline for alignment, filtering, barcode counting, and UMI counting was used to generate the multidimensional feature-barcode matrix for each replicate.

#### Batch effect correction using canonical correlation analysis

**Fig 1:**
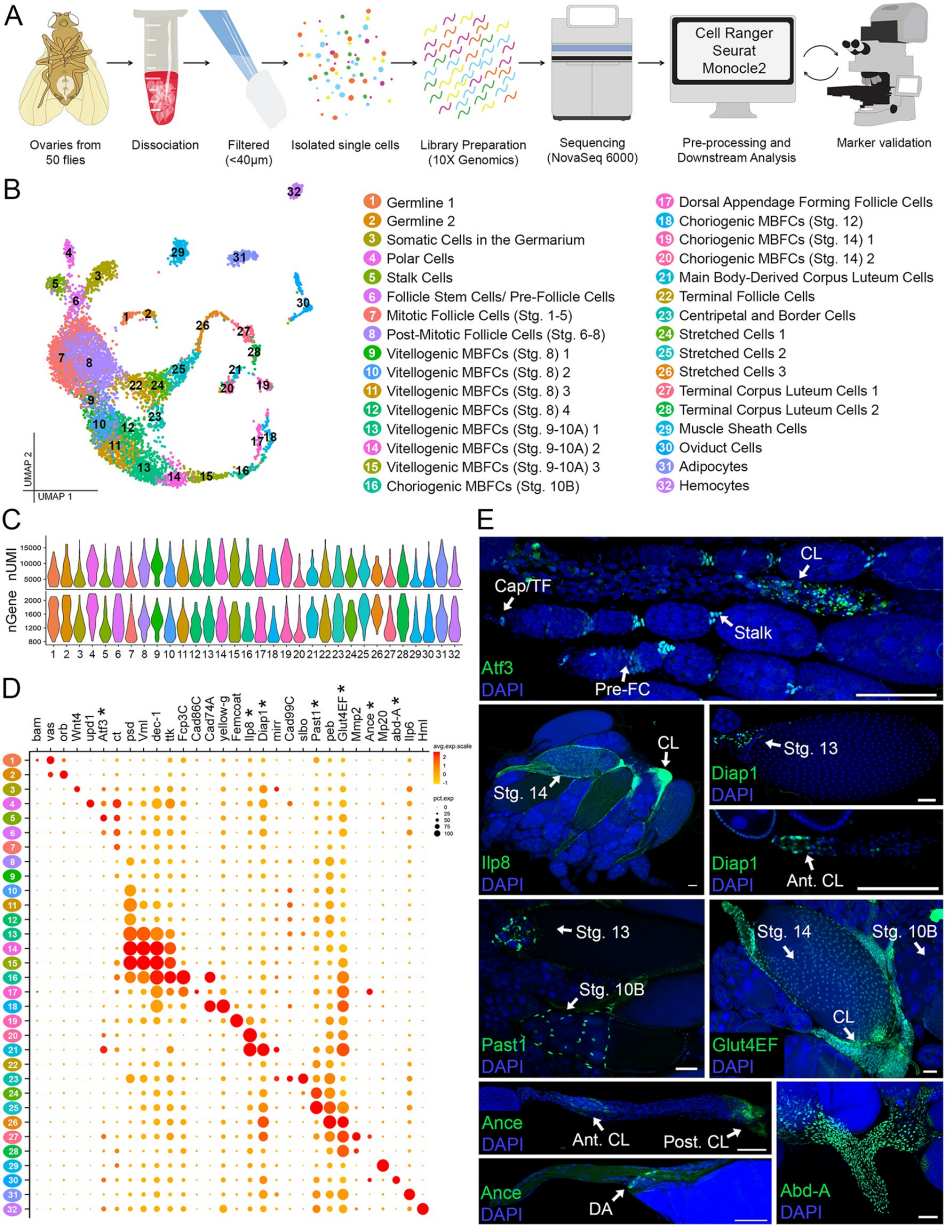
ScRNA-seq of adult *Drosophila* ovary and interconnecting tissues. (A) Illustration of the overall workflow (See also S3 Fig). (B) Annotated UMAP of 7,053 high-quality cells grouped into 32 semisupervised clusters and labeled according to cell type and stage. (C) nUMI and nGene per cluster. Clusters are numbered and colored according to cluster identity indicated in the key in panel A. (D) Dot plot of identifying marker genes (see also S1 File). Newly identified marker genes are indicated (*). Raw sequence files available from SRA repository (SRX7814226). Processed files using Cell Ranger, Seurat, and Monocle are available through GEO database (GSE146040). (E) Experimental validation of the 7 new marker genes shown in panel D. All expression (green) is marked using GFP-tagged proteins under endogenous control except Ance, marked using RFP under T2A-Gal4 control. All images are z-projections. Additional cell type and stage information is indicated. DAPI marks nuclei. Scale bar = 50 μm. Ant. CL, anterior corpus luteum cells; Cap/TF, cap and terminal filament cells; CL, corpus luteum cells; DA, dorsal appendage forming follicle cells; GFP, Green Fluorescent Protein; MBFC, main body follicle cell; nGene, number of genes; nUMI, number of unique molecular identifiers; post. CL, posterior corpus luteum cells; pre-FC, pre–follicle cells; RFP, Red Fluorescent Protein; scRNA-seq, single-cell RNA sequencing; Stg., stage; stalk, stalk cells; UMAP, Uniform Manifold Approximation Projection.

The 2 replicate data sets were then compared using canonical correlation analysis (CCA) to test for variation between the data sets caused by batch effects. Replicate 1 and 2 were aligned using 2,926 genes with the highest dispersion in both data sets, and 75 correlation vectors were used for downstream clustering. Each of the 28 clusters were comparable to the clusters in Fig 1, and a strong correlation was observed between replicate 1 and 2, indicating no significant batch effects (S1 Fig). Replicate 1 displayed a significant improvement in sampling of rarer cell types, compared to replicate 2, and was exclusively used for all downstream analyses (S1 Fig).

#### Filtration

The Cell Ranger output was used for further processing using the R package Seurat (version 2.3.4) [29, 30]. As part of this processing, reads from fragmented or multiple cells (those with less than 775 genes expressed per cell or greater than 2,200 genes and 18,000 UMIs per cell) and dead cells (greater than 1% mitochondrial gene expression) were filtered from the data set. Feature counts were log-normalized and scaled using default options. Raw read counts were used for normalization. Unwanted sources of intercellular variability were removed by regressing possible variation driven by number of UMIs and mitochondrial gene expression during data scaling (S3 Fig). Scores for the expression of an expansive list of *Drosophila* G2/M and S phase genes (S2 File) were assigned to each cell which enabled the calculation of the difference between G2/M and S phase scores, using the function CellCycleScoring. This cell-cycle score was then regressed from the downstream analysis to maintain the signals separating dividing and nondividing cells but eliminating subtle differences among proliferative cells. Based on this score, the cells were assigned a cell-cycle phase (S4 Fig). To assemble these cells into transcriptomic clusters using meaningful features, the number of random variables in our data set was reduced by obtaining sets of principal component (PC) vectors. Significant PCs were obtained by performing a principal component analysis (PCA), using 897 highly variable genes as input. The first 30 significant PCs were selected based on the Elbow method as input for Uniform Manifold Approximation and Projection (UMAP) clustering using default parameters (S3 Fig). Altogether, these preprocessing steps resulted in a primary UMAP of 12,671 cells (S2 Fig).

#### Selecting for high-quality cells using biological markers

In a single-cell data set, cells expressing markers for 2 or more cell types either indicates an intermediary cell state or the retention of doublets. Doublet signal can arise through the capture of ambient RNA in the cell suspension during library preparation along with a valid cell within a droplet or from the simultaneous capture of the fragments from 2 distinct cell types. This is a common challenge of droplet-based microfluidic library preparation methods [31]. It is crucial to minimize these contaminant signals because they decrease the precision of clustering and the fidelity of downstream analyses like pseudotemporal trajectory analysis [31, 32]. Although these can be removed using a number of in silico approaches, the reliability of these tools depends on the various assumptions that may or may not hold true in every biological context [33]. Therefore, we employed a biologically informed method instead.

The *Drosophila* ovary has been intensely studied for decades, leading to the identification and establishment of reliable cell type–and cell stage–specific expression patterns [12, 34–49]. Using this information we selected high-quality cells, based on a cutoff criteria of > *log*2 fold expression of conflicting cell-type markers in a number of distantly related cell types, for our downstream analysis (S4 File).

To ensure that we have not removed true cell types and/or intermediary cell states, we aligned this marker-cleaned data set with the original data set using CCA (S2 Fig). The 2 data sets did not lose correlation because of this cleanup process and were highly aligned, while the correlation vector with the highest correlation strength (CC1) displayed an increased dispersion across data set for the high-quality cells (compared to the original data set), thus indicating an increase in resolution of cell types. Indeed, the number of highly variable genes (limits: > 0.4 dispersion; > 0.01 and < 3 average expression) that were used for PCA, increased from 897 in the original data set to 1,075 in the high-quality data set. We also examined the expression of markers used in the cleanup process, in both original and high-quality data set and have shown the retention of all major cell types and signals less than log2FC expression (S2 Fig). Finally, the consistency between derived and expected observations of developmental trajectories provided additional validation of the quality of the cells selected for the making of this data set. The final data set of 7,053 high-quality cells and 11,782 genes was used for downstream analysis.

#### UMAP clustering analysis

Seurat was used for log-normalization and scaling of the data using default parameters. The 1,075 highly variable genes were selected as input for PCA and the first 75 PCs were selected to build the shared nearest neighbor (SNN) graph for clustering. To assemble cells into transcriptomic clusters, graph-based clustering method using the SLM algorithm [50] was performed in Seurat. We chose to plot clusters on a UMAP because this dimensionality reduction technique arranges cells in a developmental time-course in a meaningful continuum of clusters along a trajectory [51]. A number of resolution parameters, ranging from 0.5 to 6 were tested which resulted in 14 to 46 clusters. The relationship between clusters in each resolution was assessed using the R package clustree [52], based off of which a resolution of 6 was selected to obtain an initial number of 46 clusters (S2 Fig). Differentially expressed markers specific to each cluster were identified using the function FindAllMarkers (S3 File), and clusters with no unique markers were merged with their nearest neighbor after careful consideration of the differences in average expression pattern in each cluster. The final number of clusters was decided based on the uniqueness of observed and expected gene markers and the relative relationships with other clusters (S2 Fig). Cell-type identities were then assigned to each cluster using known (S1 File) and experimentally validated markers.

#### Unsupervised reclustering of cell subsets using Monocle (v2)

Smaller subsets of cells from the entire data set were selected using the SubsetData function in Seurat. These subsets were reclustered and imported into Monocle (v2) [53, 54] for further downstream analysis using the importCDS() function, with the parameter import_all set to TRUE to retain cell-type identity in Seurat for each cell. The raw UMI counts for these subsetted data sets were assumed to be distributed according to a negative binomial distribution and were normalized as recommended by the Monocle (v2) pipeline. The number of dimensions used to perform dimensionality reduction was chosen using the Elbow method (S3 Fig). The cells were clustered in an unsupervised manner using the density peak algorithm in which the number of clusters was set for an expected number of cell types (as in for early follicle-cell differentiation states) or cell states (as in mitotic-endocycle transition state, along with mitotic and endocycling follicle cells). The number of cell clusters, in case of the “germline cells” subset and the “oviduct cells” and “muscle cells” subset was chosen in an unsupervised manner based on significant rho (local density) and delta (distance of current cell to another cell of higher density) threshold values.

#### Pseudotime inference analysis and identification of lineage-specific genes of interest

Pseudotime inference analysis on known cell differentiation programs of oogenesis was performed using Monocle (v2). Cells were ordered in an unsupervised manner on a pseudotemporal vector based on genes that are differentially expressed over pseudotime between cell-type identities assigned in Seurat or cell states identified as clusters in Monocle, depending on the clustering as mentioned in the previous section. Lowly expressed aberrant genes were removed from the ordering genes. Multiple trajectories were generated by ordering the cells using different numbers of statistically significant (*q* < 0.05) genes that are expressed in a minimum number of predetermined cells, and the efficacy of the trajectories was tested with validated marker gene expression. The trajectory that reflected the most accurate cell state changes was then selected for downstream analysis. To assess transcriptional changes across a branching event, as seen in the early somatic and the polar/stalk trajectories, the function BEAM was used to analyze binary decisions of cell differentiation processes across a branch.

#### GO term enrichment analysis

Genes were selected for downstream GO term enrichment analysis from the pseudotemporal heat map by cutting the dendrogram that hierarchically clustered the genes expressed in a similar pattern across pseudotime using the R based function cutree [55]. The web-based server g:Profiler [56] and PANTHER [57] were then used for functional enrichment analysis on the genes. A user threshold of *p* = 0.05 was used for these analyses.

## Results

### ScRNA-seq identifies unique cell clusters and markers to assign cell-type identities

We generated the scRNA-seq library from a cell suspension of freshly dissected ovaries (and connected tissues) from adult female flies (Fig 1A). Following library sequencing, extensive quality control, and cell-type–specific marker validation, we recovered 7,053 high-quality cells and clustered them into 32 cell-type identities (Fig 1B, S1, S2 and S3 Figs). This data set has an average of ~7,100 UMIs and ~1,300 genes per cell, with each cell type having variable levels of mRNA content and gene expression (Fig 1C and 1D). We plotted this data set on a scale of 2 primary axes for visualization using UMAP for dimension reduction of the cell/gene expression matrix (Fig 1B). This UMAP reflects the temporal and spatial development over the entirety of oogenesis, with connected ovarian clusters forming linear trajectories from stem cells onward, while surrounding tissues with nontemporally transitioning cells (muscle sheath, oviduct, adipocytes, and hemocytes) arranged in compact and isolated clusters (Fig 1B).

Established cell-type–and stage-specific markers were used to identify the majority of the clusters (S1 File and Fig 1D). For the remaining clusters with no known markers, we assigned identity using expression patterns of at least 7 newly validated genes (Fig 1D and 1E). *Atf3* and *abd-A* were used to identify cell types such as stalk cells and oviduct cells. *Past1* was used to identify the stretched cells, and *Ilp8*, *Diap1*, *Glut4EF*, and *Ance* were used to identify late-staged follicle cells. Most of the new markers have overlapping expression in multiple cell types. For example, *Atf3*, a transcription factor involved in lipid storage [58], marks the cap and terminal filament cells in the germarium, prefollicle cells, stalk cells, and corpus luteum (CL) cells (Fig 1E). Similarly, some markers are expressed in cells across multiple timepoints, thus marking a single-cell type in several clusters. For example, *Past1*, which encodes a plasma membrane protein known to interact with Notch, marks the stretched-cell lineage in clusters 24, 25, and 26 [59]. Altogether, we were able to assign cell-type identities for all clusters and identified 6,296 genes that show significant expression in different clusters. Among them, 828 are unique markers for clusters that may be potentially specific to individual cell types (S3 File).

### The transcriptional patterns of early germline development

**Fig 2:**
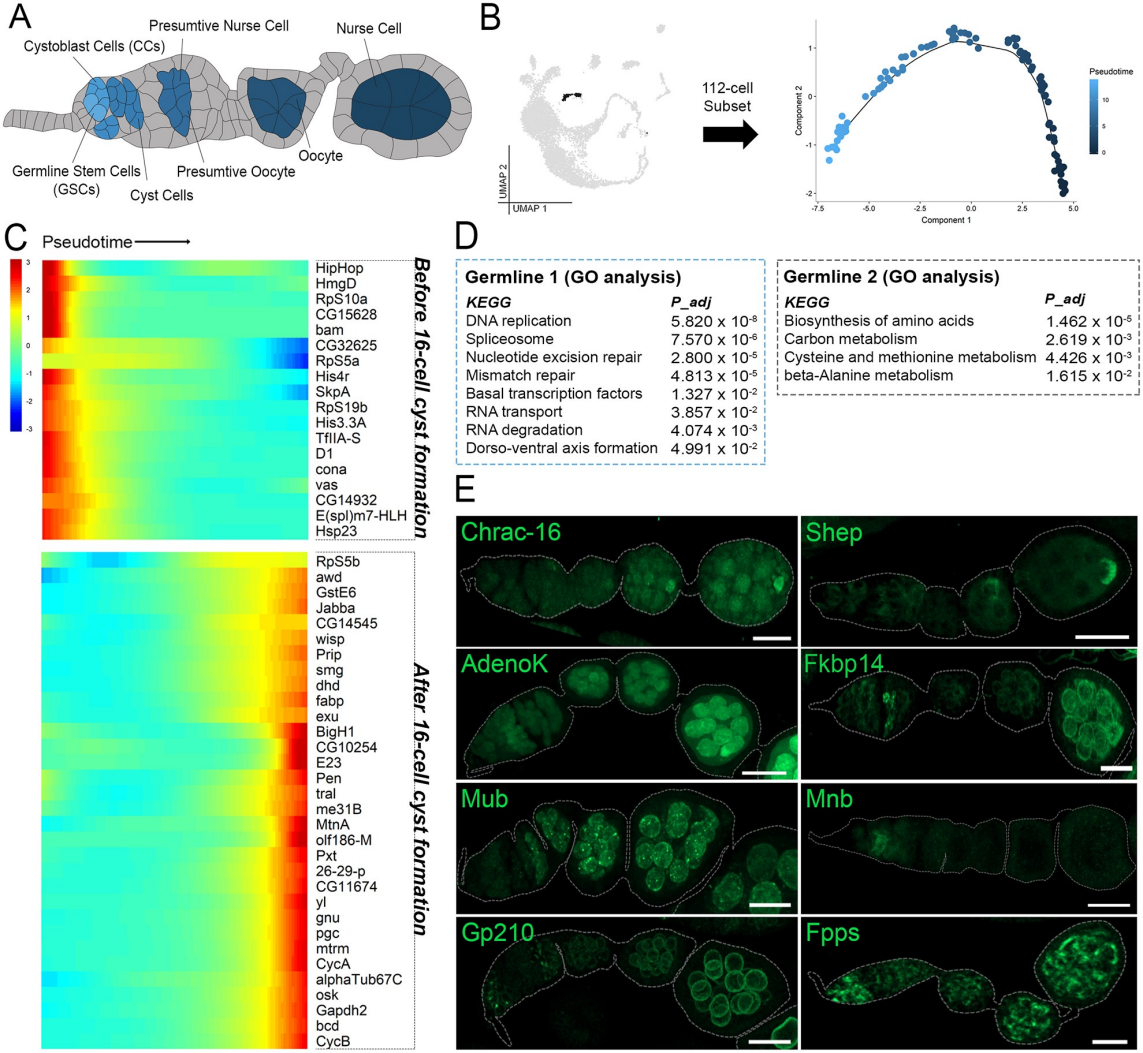
Expression patterns of germline cells during early development. (A) Illustration of early oogenesis featuring annotated germline cell types of interest (colored according to pseudotime inference in panel B and somatic cells (gray). (B) Fig 1B UMAP (gray) at left highlighting the 112-cell subset of germline clusters 1–2 (black) reclustered in Monocle for pseudotime analysis. Subset tSNE plot at right with pseudotime scale. (C) Pseudotime-ordered heat map of expression from before and after 16-cell cyst formation. Minimum expression = 5 cells; *q* < 1*e*^−5^. Data are available through GEO database (GSE146040). (D) KEGG-pathway terms and enriched for germline 1 (blue box) and germline 2 (black box) clusters. Adjusted *p*-values (*P*_adj_) are provided for each term. (G) Validation for germline expression (green) using GFP-tagged proteins under endogenous control. All images are z-projections. Ovarioles are outlined in gray. Scale bar = 20 μm. GFP, Green Fluorescent Protein; KEGG, Kyoto Encyclopedia of Genes and Genomes; tSNE, t-Distributed Stochastic Neighbor Embedding; UMAP, Uniform Manifold Approximation Projection.

Oogenesis begins in the germarium at the most anterior tip of each ovariole. There, supported by somatic niche cells, 2 to 3 germline stem cells (GSCs) produce daughter cells that move posteriorly through the niche and differentiate into cystoblast cells (CBCs) [60]. These cells undergo 4 more rounds of synchronized mitosis with incomplete cytokinesis, producing 16 interconnected germline cyst cells. One of these cells becomes a transcriptionally quiescent oocyte, whereas the others develop into nurse cells that synthesize and transport products into the oocyte through ring canals [61](Fig 2A).

The germline cells in our data set were size selected through manual filtration (see Materials and methods), resulting in a sampling from GSCs to those in mid-oogenesis. These cells form a two-cluster trajectory (Fig 1B). The Germline 1 cluster includes cells in region 1 of the germarium (marked by *bam* expression), and the Germline 2 cluster includes cells from region 2 of the germarium and onward (marked by *orb* expression) [62, 63] (Fig 1D). The formation of the 16-cell cyst occurs at the boundary of germarium region 1 and 2. To uncover the underlying expression changes occurring at this time, we arranged the 112 germline cells on a pseudotemporal axis (Fig 2B) and plotted the differentially expressed genes along pseudotime. This revealed 50 genes that are expressed significantly before or after 16-cell cyst formation (Fig 2C). Gene Ontology (GO) enrichment of KEGG-pathway terms across pseudotime revealed the broad differences in activity before and after 16-cell cyst formation. Germline 1 cells are enriched for DNA replication and repair genes, and Germline 2 cells switch to an enrichment in biosynthetic- and metabolic-pathway genes (Fig 2D). This is strikingly similar to the recent findings in a testis scRNA-seq study, which suggest an increase in mutational load in the immature germline cells of the testis and an early expression bias for DNA repair genes [64].

Selected germline-specific genes were experimentally validated and show varying expression patterns in the early stages of oogenesis (Fig 2E). Among these newly identified germline markers, specific expression of *Mnb*, a Ser/Thr protein kinase, in region 1 of the germarium and *Mub*, an mRNA splicing protein which appears only after 16-cell cyst formation, is of special interest [65, 66]. Here, we highlight other identified genes such as *Fpps* and *Gp210*, which have a dynamic temporal protein patterning in early germline cells.

### Transcriptional trajectory of early somatic differentiation

**Fig 3:**
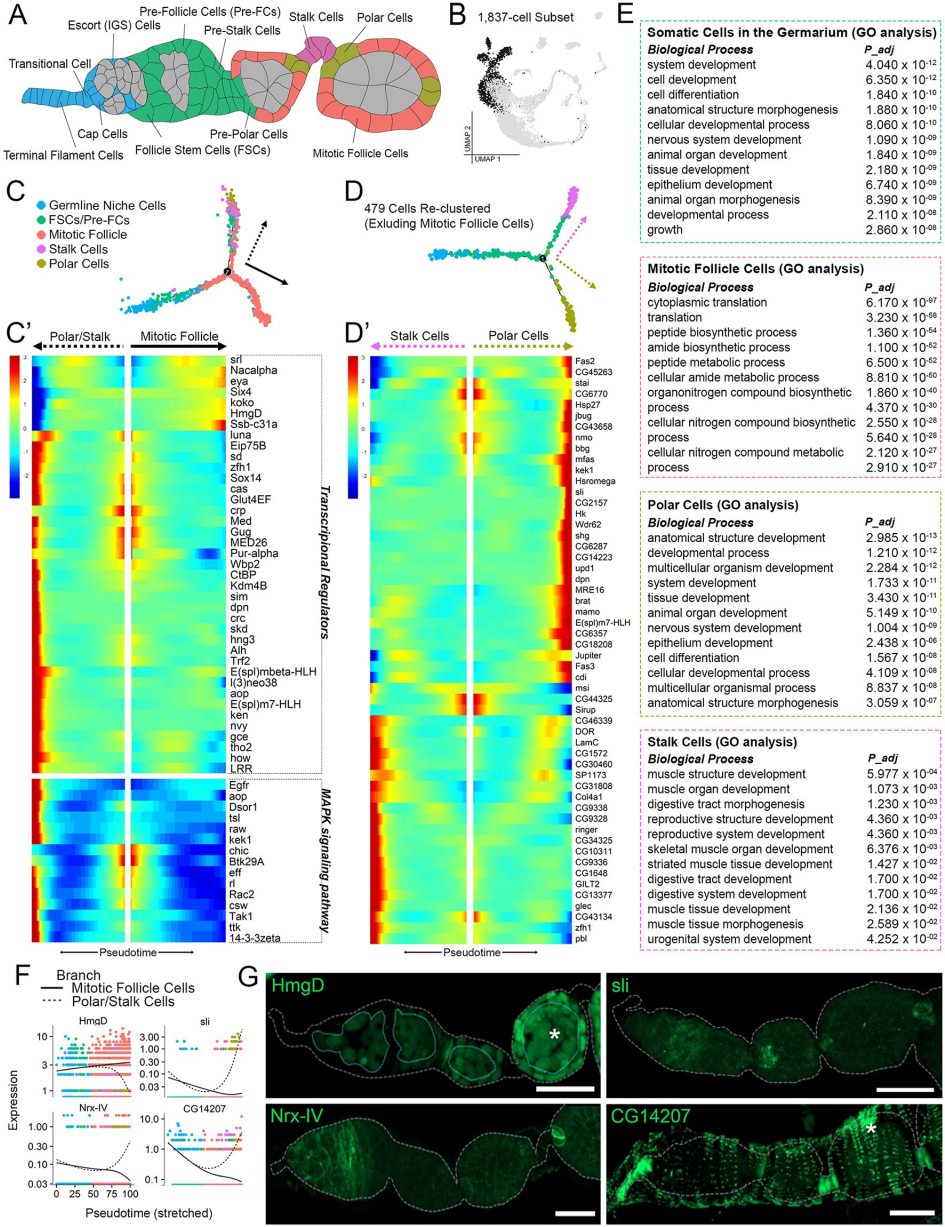
Transcription of early somatic cells during differentiation. (A) Illustration of early oogenesis featuring annotated somatic cell types of interest (colored according to identity in panel C) and germline (gray). (B) Fig 1B UMAP (gray) highlighting the 1,837-cell subset of early somatic cell clusters 3–7 (black) reclustered in Monocle for pseudotime analysis. (C) Trajectory tSNE of subset cells ordered along pseudotime. (C') Pseudotime-ordered heat map from trajectory in panel C with select genes (transcriptional regulators: GO:0140110 or PC00218, and MAPK signaling pathway: KEGG:04013) selected from expression in a minimum of 20 cells, *q* < 0.05. (D) Trajectory tSNE of the 479-cell subset (excluding mitotic follicle cells). (D') Pseudotime-ordered heatmap from trajectory in panel D. Minimum expression = 20 cells, *q* < 1*e*^−5^. (E) Enriched Biological Process terms for somatic cells in germarium cluster and mitotic follicle, polar, and stalk cell branches. Adjusted *p*-values (*P*_*adj*_) are provided for each term. (F) Expression plots of validated genes arranged along pseudotime (from trajectory in panel C) comparing the mitotic follicle cell (solid line) and polar/stalk cell (dotted line) branches. Data are available through GEO database (GSE146040). (G) Experimental validation of select genes (green) using GFP-tagged proteins under endogenous control. All images are z-projections. Ovarioles are outlined in gray. Germline outlined in top left image. Some expression is also observed in other cell types and marked with an asterisk (epithelial sheath cells in bottom right image and germline cells in top left image). Scale bar = 20 μm. GO, gene ontology; KEGG, Kyoto Encyclopedia of Genes and Genomes; MAPK, Mitogen-Activated Protein Kinase; PC, principal component; tSNE, t-Distributed Stochastic Neighbor Embedding; UMAP, Uniform Manifold Approximation Projection.

The anterior region of the germarium houses somatic cells that include 8 to 10 terminal filament cells, a pair of cap cells, and the escort, or inner germarium sheath (IGS), cells. These collectively form the germline stem cell niche [2, 5] (Fig 3A). The follicle stem cells (FSCs) reside between germarium regions 1b and 2b [67]. It is thought that typically 2 FSCs are active in each germarium; however, the most recent report indicates that this number could fluctuate between 1 and 4. [6]. The FSCs produce daughters, pre–follicle cells (pre-FCs), which envelope the germline cyst cells, forming an egg chamber. As egg chambers pinch off from the germarium, preFCs at the 2 poles assume polar cell fate upon Notch activation. The anterior polar cells then promote the specification of the stalk cells through Janus Kinase/Signal Transducer and Activator of Transcription (JAK/STAT) signaling [7]. The polar and stalk cells cease division upon differentiation while the other follicle cells remain mitotically active [68].

Because of the unsupervised nature of our clustering, the somatic cells in the germarium are clustered together (Fig 1B). This suggests a common transcriptomic signature that may be a response to the shared stem cell niche signaling. GO analysis for this group revealed an unexpected enrichment of nervous system development related genes, among more general development- and morphogenesis-related genes (Fig 3E).

To determine the transcriptional trajectory during early somatic differentiation, we arranged the 1,837-cell subset from clusters containing somatic cells of the germarium, polar cells, stalk cells, and mitotic follicle cells on a pseudotemporal axis (Fig 3B and 3C). This pseudotemporal trajectory establishes a divergence of the follicle-cell lineage after FSC/pre-FC differentiation, because the branch for mitotic follicle cells separates out from a common branch for the polar/stalk cell lineage (Fig 3C). This trajectory is consistent with the notion that polar and stalk cells share a common precursor stage and share expression of certain commonly up-regulated transcription factors as shown in other studies [69, 70].

Considering the importance of transcriptional regulation in differentiation, we analyzed the temporal patterns of highly expressed genes selected for their function as either transcription regulators (GO:0140110) or transcription factors (PC00218; Fig 3C'). Plotting these genes across pseudotime revealed that the polar/stalk cell fates are transcriptionally dynamic, involving genes from many signaling pathways. We highlighted the genes involved in the MAPK pathway (Fig 3C'). Fewer transcription factors are expressed in the mitotic follicle-cell lineage (Fig 3C'). Among them are the chromatin remodeling protein HmgD and its physical interactor, Nac*α*, suggesting a role of epigenetic regulation in the proliferative effort of these cells [71, 72] (Fig 3C', 3F and 3G). The mitotic follicle-cell lineage also shows a differential enrichment of ribosomal genes (*KEGG*: 03010, *P*_*adj*_ = 2.20*e*^−49^), probably to support the up-regulation of biosynthetic processes to sustain rapid proliferation (Fig 3E).

### Fate decisions during polar and stalk cell differentiation

To characterize the fate separation between polar and stalk cells, we excluded the mitotic follicle cells from further analysis. The resulting 479 cells were then ordered once again along a pseudotemporal axis (Fig 3D). The resulting trajectory shows that the polar cells differentiate earlier than the stalk cells, which is consistent with the evidence that chemical cues from polar cells initiate stalk cell differentiation [7, 69]. To further identify genes that regulate polar and stalk cell differentiation, we plotted the most significant (*q* < 1*e*^−5^) differentially expressed genes between the 2 fates (Fig 3D'). GO analysis of biological functions in the polar cell branch revealed a remarkable number of genes involved in processes related to nervous system development, neurogenesis, and neuron differentiation, similar to neuron-related expression in somatic cells of the germarium (Fig 3E).

Many such genes (e.g., *Fas2*, *bbg*, *kek1*, *sli*, *shg*, *brat*, *Fas3*, and *CG18208*) produce junction proteins (*CG*: 0005911, *P*_*adj*_ = 5.563*e*^−4^) or proteins at the cell periphery (*CG*: 007194, *P*_*adj*_ = 2.568*e*^−2^; Fig 3D'). We validated the expression of *sli*, a novel polar cell marker, which is a secreted ligand for the Slit/Robo signaling pathway (Fig 3F and 3G). Another validated polar cell marker, *Nrx-IV*, is also associated with this pathway [73] (Fig 3F and 3G). In addition to axon guidance in developing neurons, Slit/Robo has been implicated in the regulation of tissue barriers [74], which is consistent with the observation that polar cells are terminally differentiated barriers between each egg chamber unit and connecting stalk cells [75].

GO term analysis of stalk cell specific genes indicates a highly significant (*q* < 1*e*^−5^) up-regulation of extracellular matrix genes (e.g., *Col4a1*, *LanB1*, and *vkg*) and cytoskeletal genes (e.g., *LamC* and *βTub56D*) that are also involved in muscle structure development (Fig 3D' and 3E). Supporting this finding, we found a novel stalk cell marker *CG14207* that is also expressed in epithelial muscle sheath (Fig 3F and 3G). Its human homolog, HspB8, interacts with Stv at the muscle sarcomere as part of a chaperone complex required for muscle Z-disc maintenance [76].

### Catalytic genes up-regulated during mitosis-endocycle transition of follicle cells

**Fig 4:**
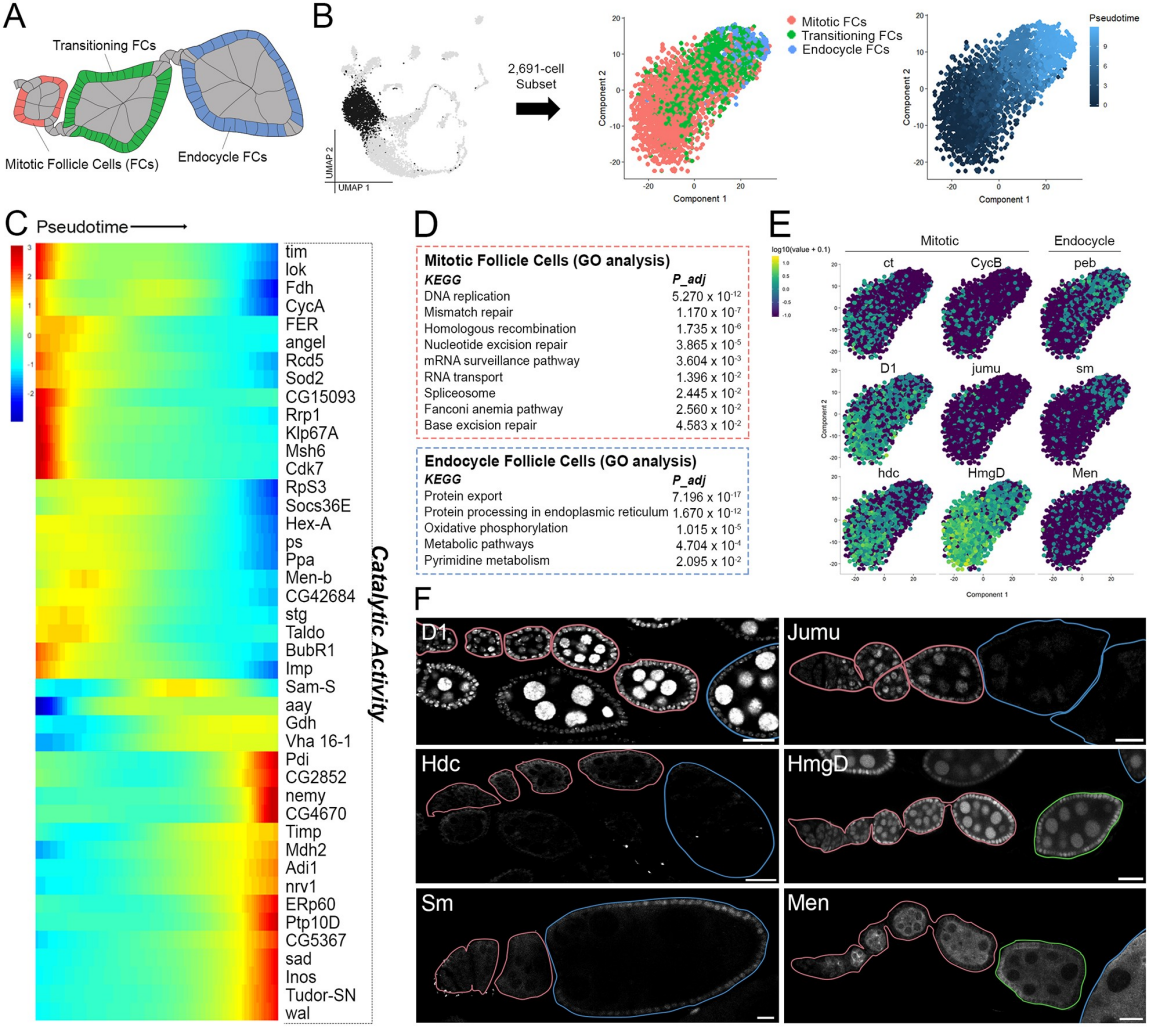
Gene expression during M/E transition in follicle cells. (A) Illustration of follicle cells of interest during M/E switch (colored according to their cluster color in panel B) with all other cells in gray. (B) Fig 1B UMAP (gray) highlighting the 2,691-cell subset of early to mid-staged follicle cells from clusters 7 and 8 (black) reclustered in Monocle for pseudotime analysis (left). Subset tSNE with cluster annotation informed by ct, CycB, and peb marker expression shown in panel E (center). Subset tSNE with pseudotime colors (right). (C) Pseudotime-ordered heat map of highly expressed genes grouped by catalytic activity (GO:0003824). Minimum expression = 20 cells; *q* = 0.05. (D) KEGG-pathway terms enriched in mitotic and endocycling follicle cells (early and late expressing genes respectively from panel C). (E) Feature plots for select genes showing differential patterning in either mitotic or endocycle follicle cells. Top row genes (*ct*, *CycB*, and *peb*) are known markers. The others are newly identified. Data are available through GEO database (GSE146040). (F) Experimental validations for newly identified M/E switch markers (white) using GFP-tagged proteins under endogenous control. Ovarioles are outlined and colored according to stage: germarium and mitotic stages (pink), transitional stage (green), and endocycle stages (blue). All images are a z-slice through the center of each ovariole. Scale bar = 20 μm. GO, gene ontology; KEGG, Kyoto Encyclopedia of Genes and Genomes; M/E, mitosis-endocycle; tSNE, t-Distributed Stochastic Neighbor Embedding.

The transition between early and middle oogenesis (stages 6–7) occurs when the germline cells up-regulate the ligand Dl, activating Notch signaling in the follicle cells, which initiates a mitosis-endocycle (M/E) switch [77] (Fig 4A).

To understand the regulation of the M/E switch at the single-cell level, we reclustered the 2,691 follicle cells from clusters 7, 8, and 9 and arranged them across pseudotime (Fig 4B). Known Notch targets were used to validate cluster identity: *ct* and *CycB* in mitotic cells, *peb* in endocycling cells [78, 79], and all 3 in transitioning cells (Fig 4E). Pseudotime analysis revealed a linear arrangement for genes that change expression levels during the M/E switch. We validated some of these newly identified genes. For example, *D1*, *jumu*, and *hdc* are down-regulated, whereas *Men* and *sm* are up-regulated in postmitotic follicle cells (Fig 4F). The NADP(Nicotinamide adenine dinucleotide phosphate)[+] reducing enzyme, *Men*, is up-regulated significantly in the anterior follicle cells and has a membrane localization. *Sm*, a member of the heterogeneous ribonucleoprotein complex, is of special interest given its ability to regulate Notch activity during wing development [80]. Its enrichment in endocycling follicle cells suggests a potential role for sm in Notch-mediated M/E switch. Noticeably, upon GO term enrichment analysis of all significantly expressed genes that change as a function of pseudotime during the M/E switch, we found 43 genes with catalytic activity (GO:0003824; Fig 4C). Enriched KEGG-pathway–related terms reveal an expression bias for proliferation and DNA repair associated genes in mitotic follicle cells, whereas endocycling cells express protein-processing and metabolic genes (Fig 4D).

### Transcriptomic divergence of mid-staged follicle cells with subsequent convergence

**Fig 5:**
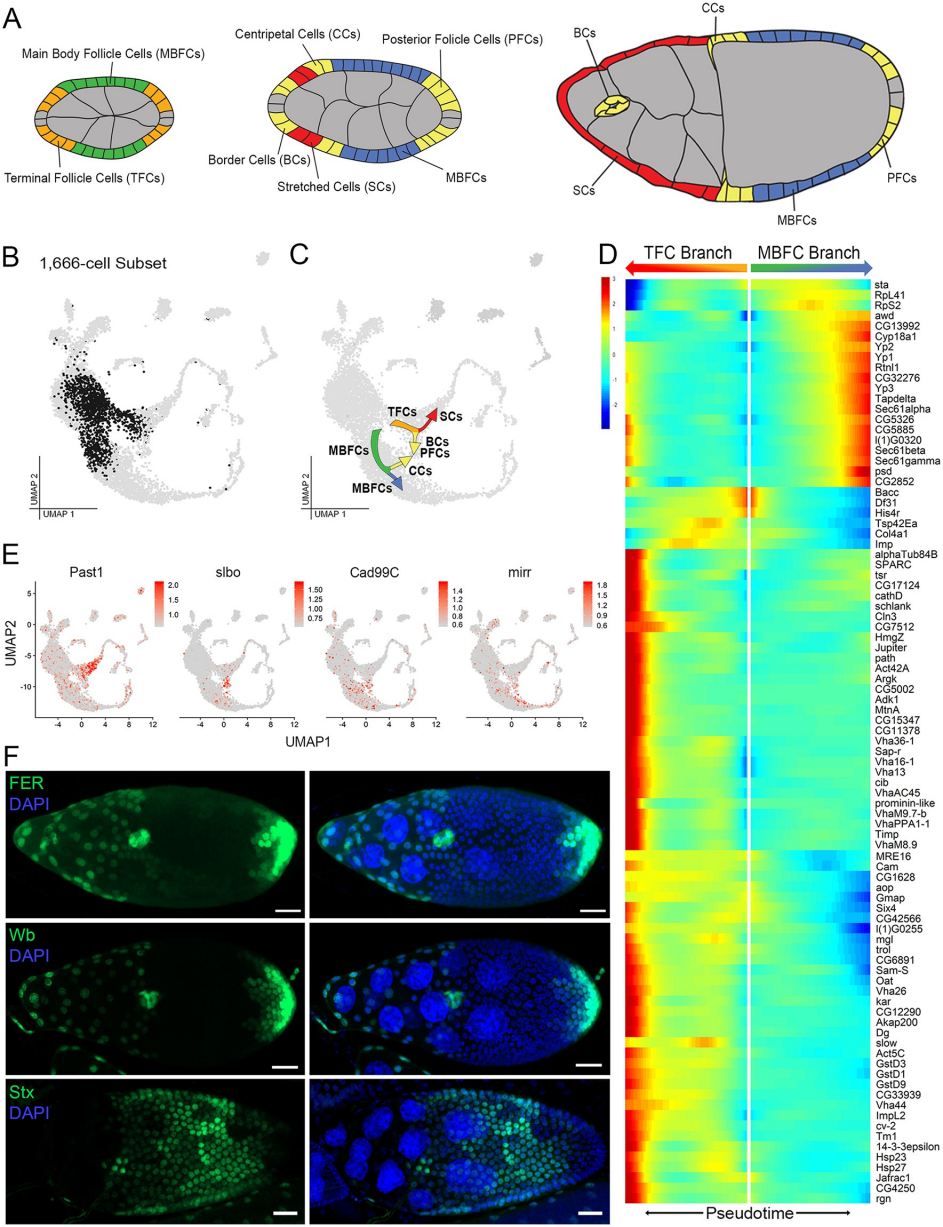
Transcriptional divergence of TFCs during symmetry breaking with subsequent convergence of slbo-expressing cells. (A) Illustration of annotated follicle-cell types during symmetry breaking and differentiation (colored by type) with all other cell types shown in gray. Stalk cells not shown. (B) Fig 1B UMAP (gray) highlighting the 1,666-cell subset of mid-staged follicle cells in clusters 8–10 and 22 (black) reclustered in Monocle for pseudotime analysis. (C) Fig 1B UMAP (gray) annotated with cell-type lineage information based on markers in panel E. (D). Pseudotime-ordered heat map of gene expression during the TFC and MBFC branching in panel C. Minimum expression = 20 cells; *q* < 1*e*^−20^. (E). Feature plots of marker genes used for identification in panel C. Past1 = SCs, slbo = BCs, PFCs, and CCs, Cad99C = CCs, mirr = MBFCs. Data are available through GEO database (GSE146040). (F) Experimental validation of select gene expression (green) in cells after symmetry breaking (not shown in heatmap in panel D). All lines express GFP under T2A-Gal4 control for each gene. *FER* and *wb* are expressed in SCs, BCs, and PFCs. *Stx* is expressed in MBFCs. All images are z-projections. DAPI marks nuclei. Scale bar = 20 μm. BC, border cell; CC, centripetal cell; GFP, Green Fluorescent Protein; MBFC, main body follicle cell; PFC, polar follicle cell; SC, stretched cell; TFC, terminal follicle cell; UMAP, Uniform Manifold Approximation Projection.

During early oogenesis, access to morphogen signals from polar cells are restricted to the nearby terminal follicle cells (TFCs) on either end of the egg chamber [81]. The posterior TFCs receive a signal from the oocyte to activate epidermal growth factor receptor (EGFR) signaling around stage 6, marking a symmetry breaking event in follicle cells. Cells at the anterior terminal further specify into border, stretched, and centripetal cells and undergo massive morphological changes during stages 9–10B [13] (Fig 5A).

Our data set shows an unanticipated transcriptomic divergence for postmitotic follicle cells, which provides a transcriptional basis for follicular symmetry breaking (Fig 1B). To identify the fate assumed by the cells in each resulting branch, we validated the expression of known markers at this stage and also novel markers uncovered from reclustering 1,666 cells of this stage (Fig 5B). The MBFC branch was identified using *mirr* and *Cad99C* expression [82, 83]. And the TFC branch identity was validated by the expression of newly identified anterior terminal cell marker, *Past1* (Fig 5E).

We took the 1,666-cell subset of follicle cells during symmetry breaking and arranged them on a pseudotemporal axis (Fig 5B). Then we performed a GO term enrichment analysis of the differentially expressed genes at the branching point between MBFC and TFC fate. The MBFC fate shows an enrichment of genes in protein export (*KEGG*: 03060, *P*_*adj*_ = 8.55*e*^−20^) and protein processing in the endoplasmic reticulum (*KEGG*: 04141, *P*_*adj*_ = 1.13*e*^−17^); whereas the TFC fate has an enrichment of genes in endocytosis (*KEGG*04144, *P*_*adj*_ = 1.70*e*^−9^), proteasome (*KEGG*: 03050, *P*_*adj*_ = 3.46 − 7), phagosome (*KEGG*; 04145, *P*_*adj*_ = 6.97*e*^−6^), glutathione metabolism (*KEGG*: 00480, *P*_*adj*_ = 2.09*e*^−2^), oxidative phosphorylation (*KEGG*00190, *P*_*adj*_ = 2.01*e*^−2^), and Hippo pathway (*KEGG*: 04391*P*_*adj*_ = 3.95*e*^−2^). The 89 genes that show significant differences between these 2 branches along pseudotime are highlighted in a heat map (Fig 5D). Many genes are differentially up-regulated in these 2 branches much later in pseudotime.

We also identified novel genes showing expression that coincides with the symmetry breaking process (Fig 5F). These include *FER* and *wb*, which regulate cytoskeletal rearrangement, cell adhesion, and extracellular components. These genes may participate in cell shape changes necessary for border cell migration and/or SC flattening [84, 85]. On the other hand, MBFC-specific expression of *stx* is interesting because it is involved with the proteasomal degradation regulating Polycomb (Pc) stability [86]. Maintenance of MBFC fate through regulation of chromatin modifiers is an attractive direction that merits further research.

### Expression profiles of migrating border and centripetal cells

**Fig 6:**
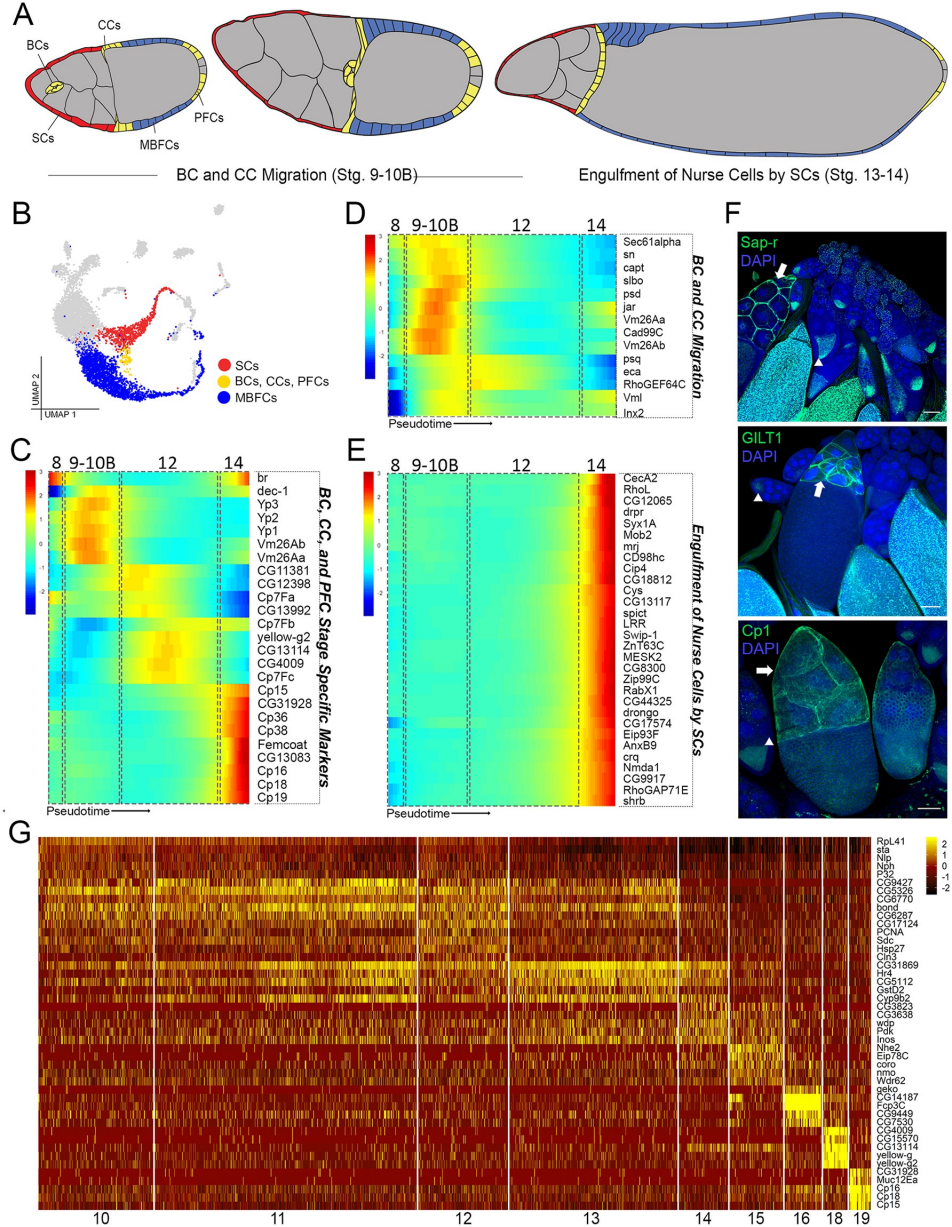
Gene expression in follicle cells during migration, nurse-cell engulfment, and vitellogenesis. (A) Illustration of annotated follicle cells of interest (colored according to UMAP in panel B) with all other cell types in gray. Stalk cells not shown. (B) Fig 1B UMAP (gray) highlighting the mid–late stage follicle-cell subsets reclustered in Monocle for pseudotime analysis. TFCs and SCs subset = 798 cells from clusters 22–26 (red). BCs, CCs, and PFCs subset = 193 cells from cluster 23 (yellow). MBFCs subset = 1,988 cells from clusters 10–16 and 18–19 (blue). (C) Pseudotime-ordered heat map of stage 8–14 specific markers from red and yellow subsets from panel B. Estimated stage boundaries (dotted boxes) are superimposed on the heat map. (D) Pseudotime-ordered heat map of genes during stage 9–10B (in cells from yellow and red subsets) with epithelial development genes (*GO*: 0060429, *P*_*adj*_ = 1.101*e*^−5^) specifically highlighted. Minimum expression = 100 cells; *q* < 0.05. (E) Pseudotime-ordered heatmap of red and yellow subset genes in stage 14 highlighting the 30/79 genes also expressed in hemocyte cluster 32 from Fig 1B. Minimum expression = 50 cells; *q* < 0.05. Data are available through GEO database (GSE146040). (F) Experimental validation for 3 highly expressed genes in SCs (not shown in the heat map in panel E) using GFP-tagged proteins under endogenous control. Arrows point to SCs and arrowheads point to additional expression in oocytes. All images are a single z-slice through the center of egg chambers. DAPI marks nuclei. Scale bar = 20 μm. (G) Heat map of top 5 highly expressed genes per cluster for the blue subset (clusters 10–16, 18–19 from Fig 1B). BC, border cell; CC, centripetal cell; GFP, Green Fluorescent Protein; MBFC, main body follicle cell; PFC, posterior follicle cell; SC, stretch cell; TFC, terminal follicle cell; UMAP, Uniform Manifold Approximation Projection.

During stages 9–10B, specialized subsets of TFCs transition from a stationary to migratory state. These include the border cells, which delaminate from the epithelium and move through the nurse cells to reach the oocyte. There, they meet the centripetal cells which migrate inward to cover the anterior end of the oocyte (Fig 6A).

In our plot, we found that the TFC and MBFC branches converge to form a distinct cluster marked by *slbo*, which is expressed in migrating border and centripetal cells [14] (Fig 5C). To examine the transcriptomic signature of these migratory cells, we first used known stage 8–14 markers [11, 12] to set stage boundaries for the TFC branch (Fig 6B and 6C). This boundary was then used to select gene expression specifically during cell migration. We highlighted 14 representative genes involved in epithelial development (*GO*: 0060429, *P*_*adj*_ = 1.101*e*^−5^), the highly enriched GO term in this cluster. These include markers for border cell migration, such as *sn*, *jar*, and *Inx2* [15, 87–89]. We also detected in this cluster the expression of *Cad99C*, which has been reported in several MBFCs and anterior-migrating centripetal cells [83]. These known markers confirm the correct selection of migrating cell types. This cluster also show expression of other stage 9–10B markers, such as vitelline membrane-related genes: *psd*, *Vm26Aa*, *Vm26Ab*, and *Vml* [12, 41, 44]. With the confidence in our selection of stage 9–10B migrating cells, we identified additional genes such as protein transmembrane transporter *Sec61α*, actin binding protein *capt*, cargo receptor *eca*, and Rho guanyl-nucleotide exchange factor *RhoGEF64C*, which may contribute to different aspects of the cell migration process [71, 90–93] (Fig 6D).

### SCs share the transcriptional signature with hemocytes as they engulf nurse cells

During the final stages of oogenesis (stages 13–14), after the nurse cells transfer their cytoplasm into the oocyte, the remaining nuclei and cellular contents are removed by the SCs. This phagocytic activity of SCs is reminiscent of the response of hemocytes upon infection [94]. To determine whether genes expressed in the SC cluster are also expressed in hemocytes, we examined the stage 13–14 specific genes identified from the pseudotemporally arranged 798-cell subset of the TFC branch. We identified 11 genes in this cluster (*LRR*, *PGRP-SD*, *Irbp18*, *PGRP-LA*, *Hsp26*, *trio*, *bwa*, *Hsp67Bc*, *CecA2*, *Hsp27*, and *Hsp23*) categorized by their involvement in immune system process (GO:0002376). We also compared genes enriched in the SCs with those in the hemocyte cluster and found 79 genes in common. Of these, 30 genes with the highest expression are shown in a heatmap ordered across pseudotime (Fig 6E). Some immune genes have been identified previously in nurse-cell engulfment, such as the phagocytic gene *drpr* and a scavenger receptor gene *crq*, confirming sampling of the correct developmental time point for analysis [94, 95]. The newly identified genes in the SC cluster fall into 6 general categories of activity: endocytosis/vesicle mediated transport (*Syx1A*, *RabX1*, *AnxB9*, and *shrb*), antibacterial/immune response (*CecA1* and *LRR*), morphogenesis (*Mob2*, *CG44325*, *RhoGAP71E*, and *RhoL*), catalytic/metabolic (*CG12065*, *Cip4*, and *Nmda1*), lipid binding (*Cip4* and *Gdap2*), and metal ion transport, especially zinc and magnesium (*spict*, *Swip-1*, *ZnT63C*, and *Zip99C*). In addition, we validated 3 new SC genes (Fig 6F), which are also expressed in hemocytes: a proteolytic enzyme, *Cp1*, involved in cellular catabolism, an oxidation-reduction enzyme, *GILT1*, involved in bacterial response, and *Sap-r*, a lysosomal lipid storage homeostasis gene with known expression in embryonic hemocytes [96–98]. Together, these findings suggest that SCs and hemocytes share transcriptomic signatures required for apoptotic cell clearance, reinforcing their role as “amateur” phagocytes at this stage of development [99].

### Gene expression of vitellogenic MBFCs

The clusters for the MBFCs show an enrichment of genes that facilitate vitellogenesis (stages 8–14) and egg shell formation (stages 10–14; Fig 1D). We further analyzed the clusters of the MBFC clusters and found highly variable gene expression patterns (Fig 6B and 6G). Genes enriched in clusters 10–13, presumably consisting of stage 8–10A MBFCs, include histone binding protein-coding genes such as *Nlp*, *Nph*, and *P32*, which have been shown to cooperate in the post fertilization regulation of sperm chromatin [100]. Starting in cluster 16, marked by the stage 10B specific marker *Fcp3C*, chorion-related genes such as CG14187, acid phosphatase *CG9449*, and signaling receptor *CG7530* show an up-regulation. Stage 12 and 14 follicle cells (clusters 18 and 19, respectively) express well-known markers involved with chorion production (e.g., *CG4009*, *CG15570*, *CG13114*, *yellow-g*, *yellow-g2*, *CG31928*, *Muc12Ea*, *Cp16*, *Cp18*, and *Cp15*) [12] (Fig 6G).

### Cellular heterogeneity and markers in the CL

In a recent study, a final follicle-cell transition was identified from oogenesis to ovulation [20]. Ovulation occurs when a mature egg sheds the follicle-cell layer and exits the ovary on its way to be fertilized, following *Mmp2*-dependent rupture of posterior follicle cells. The follicle-cell layer, devoid of the egg as a substrate, remains in the ovary and develops into a CL, similar to ovulation in mammals [20, 101].

As mentioned previously, we validated a number of genes such as *Ance*, *Diap1*, *Ilp8*, and *Glut4EF*, which all show expression in the CL cell clusters (Fig 1E). The insulin-like peptide, *Ilp8*, involved in coordinating developmental timing, is greatly up-regulated in stage 14 follicle cells and persists in CL cells [102]. The caspase binding enzyme, *Diap1*, is highly expressed in late-stage (11–14) anterior follicle cells and persists in anterior CL cells [103]. The transcription factor, *Glut4EF*, shows increased expression from stage 10B MBFCs and reaches the highest expression level in stage 14 follicle cells and CL cells [104]. Expression of *Ance*, a gene producing an extracellular metallopeptidase, is specific to the terminal CL cells, as well as subsets of oviduct and dorsal appendage forming cells [105].

**Fig 7:**
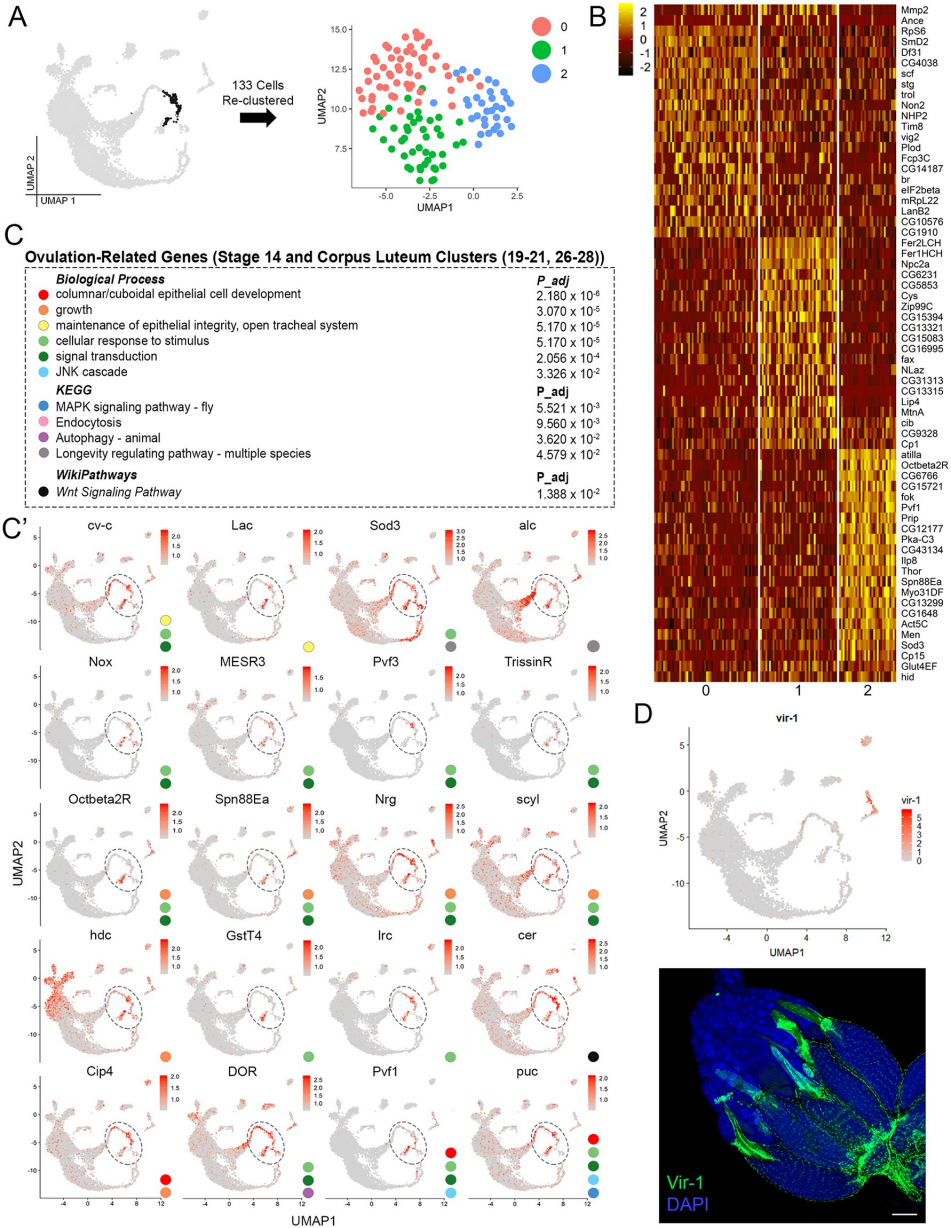
Ovulation-related genes in pre-CL cells and heterogeneity of the CL. (A) Fig 1B UMAP (gray) highlighting the 133-cell subset of CL cell clusters 21 and 27–28 (black) reclustered at right. (B) Heatmap of top 20 genes per cluster (including validated markers *Mmp2*, *Ance*, and *Glut4EF* in Fig 1) from subset plot in panel A. (C) GO analysis of enriched, ovulation-related genes from all stage 14 follicle cell (Stage 14 FC) also called pre-CL clusters (19–20, 26) and CL clusters (21, 27–28). (C') Feature plots of select ovulation-related genes in panel C. Colored circles indicate the GO term in panel C that each gene belongs to. Dotted ovals mark pre-CL and CL regions of interest. Data are available through GEO database (GSE146040). (D) Experimental validation of *vir-1* (green) marked using RFP expression under T2A-Gal4 control. Expression indicated in stage 14 follicle cells before ovulation (arrow: top image) and in CL after ovulation (arrow: bottom image). Additional expression in oviduct cells indicated (*). Both images are z-projections of an entire ovary. DAPI marks nuclei. Scale bar = 100 μm. CL, corpus luteum; FC, follicle cell; GO, gene ontology; RFP, Red Fluorescent Protein; UMAP, Uniform Manifold Approximation Projection.

To explore cellular and transcriptomic heterogeneity of the CL, we reclustered the 133-cell subset of CL cells from original clusters 21, 27, and 28 (Fig 6A). The cells reclustered into 3 groups, labeled clusters 0, 1, and 2 (Fig 6B). Both *Mmp2* and *Ance* are expressed in clusters 0 and 1, indicating that they are composed of the TFCs of the CL, likely at different time points (Fig 7B). This also indicates that the anterior and posterior CL might be transcriptionally similar. Cluster 2 most likely represents the cells derived from MBFCs as they express genes such as *Ilp8* and *Glut4EF* that are expressed throughout the CL (Fig 7B). These results suggest cellular heterogeneity in the CL with specific functions of cells in different regions.

### A transcriptomic switch from oogenesis-to-ovulation regulation in pre-CL cells

As stated previously, CL-enriched genes, *Ilp8* and *Glut4EF*, begin their peak expression in late stage-14 follicle cells. A third, viral-response gene, *vir-1*, displays a similar pattern of sudden up-regulation in stage 14 follicle cells and continued expression in CL cells after ovulation [106] (Fig 7D). Because of this shared expression timing of non–egg-shell–related genes, we considered the stage-14 clusters from the SC and MBFC lineage as a “pre-CL” and compared genes shared by these cells and those in the CL to gain insight into potential ovulation-related genes at the end of oogenesis.

GO term enrichment analysis of the genes identified using this method are involved in various biological processes, such as columnar/cuboidal epithelial cell development, growth, maintenance of epithelial integrity, cellular response to stimulus, signal transduction, and JNK cascade. Several key developmental pathways such as MAPK, endocytosis, autophagy, longevity, and Wnt signaling are also enriched (Fig 7C). One of these genes, *Nox*, an NADPH oxidase, is expressed in mature follicle cells and is required for ovulation to occur [107] and has also been shown to be essential for ovulation regulation in the oviduct [108]. Another gene identified as essential for ovulation in the oviduct [109], *Octβ2R*, encoding an octopamine receptor, was also identified here in the mature follicle/pre-CL cells. Like *Nox* and *Octβ2R*, we observed many other genes that were expressed in both the mature follicle/pre-CL cells and oviduct cells. One such antiviral gene, *vir-1* [110], has been experimentally validated to ensure expression in both mature follicle cells, CL cells, and oviduct cells (Fig 7C' and 7D).

## Discussion

In this study, we used scRNA-seq to survey the expression profiles of cells from the adult *Drosophila* ovary. Using this approach, we recovered high-quality cells through removing contaminants with conflicting marker expression and experimentally validating the identity of clusters using new markers identified in the data set. During dissection, instead of mechanically separating intimately connected tissues (i.e., muscle sheath, hemocytes, oviduct, and fat body) from the ovary, we chose to leave them attached, including them in the data set. Separating cells from different tissues in this way prevented damage to the ovarian cell types of interest and improved feature selection in downstream analysis. This approach allowed the clustering of all possible cell types that are physically connected to the ovary, thus taking account of cells that otherwise would have appeared as unknown contaminants. This enabled stringent fidelity assessment and inclusion of only high-quality cells with compatible biological markers.

With a special focus on the most abundant ovarian cell type, the follicle cells, we identified their entire spatiotemporal trajectory from the stem cell niche to the CL. Using in silico subset analyses, we identified the transcriptomic basis for early differentiation of polar and stalk cells from the MBFCs, mitosis-to-endocycle switch, and follicular symmetry breaking. We also identified transcriptomic signatures of different follicle-cell groups that carry out important developmental functions such as migration, engulfment of nurse cells, and egg-shell formation. Remarkably, the data set not only reveals a novel split in the transcriptome during symmetry breaking but also a convergence of late-stage follicle cells as they form the CL. During this convergence, we identified new ovulation-related genes in late-stage follicle cells (termed pre-CL) as they undergo the developmental switch from oogenesis-to-ovulation regulation, which was recently identified [20]

An unexpected advantage of this approach is the ability to analyze the relationship between ovarian and nonovarian cell types, which show functional convergence between cells of different tissues. For example, the nurse cell engulfing SCs express genes shared by the hemocytes. Whereas some immune-related genes have been described in these “amateur” phagocytes [99], other morphology-regulating genes shared with hemocytes have not yet been identified. This introduces an interesting possibility that aspects of SC and hemocyte morphology may be essential for the engulfment of cellular material, which necessitates further research. Additionally, cells in the CL possess a transcriptomic signature that has overlapping genes expressed in the oviduct cells and hemocytes, indicating a potential shared function or interaction between these cell types in regulating ovulation. This is consistent with reports in mammals that the CL functions as an endocrine body for control of reproductive timing [111, 112] and has signaling cross-talk with macrophages [113, 114]. Overall, our study provides a broad perspective of functional relatedness among cell types regulating oogenesis and ovulation. The convergence of such transcriptional “tool kits” between developmentally unrelated cell types is an emerging theme identified using this diverse data set. This is consistent with the recent discovery of correlated gene modules (CGM), clusters of intercorrelated genes that function together [115]. Curating information on genes that define these overlapping functions will not only help further our current understanding of GO but also identify unique genes that may have differential functions in specific cell types.

As it stands, a major challenge in the scRNA-seq field is the current lack of gold standard practices for sample preparation and analyses. There is also a lack of unanimity on how and when to incorporate replicates [116, 117]. It should be noted that in our study, although information from both biological replicates were used to eliminate batch effects, the rest of the analyses were restricted to only one replicate. Although this may be perceived as a potential limitation, our confidence in the validity of information provided by this single-replicate data set comes from rigorous experimental validation and consistent expression of canonical markers identified through decades of previous work in the field. As more ovarian data sets are published, there will be an opportunity to compare and contrast different analysis approaches. One such data set, focusing mainly on early ovarian somatic cells in *Drosophila*, was recently reported in a preprint article [118]. Despite the differences in sampling, sample preparation, and analysis methods, they report similar cell groupings and comparable marker expression patterns. Additional comparisons to the scRNA-seq analysis of the developing ovary in the *Drosophila* larvae [119] may yield important information with respect to the follicle stem cell precursors identified there and provide a deeper insight into cellular and transcriptomic changes that occur during metamorphosis.

Taken together, our study provides a novel perspective of oogenesis, identifies cell-type and stage markers, and reveals functional convergence in expression between ovarian and nonovarian cell types. Additionally, it is now possible to use this single-cell data set to better understand the intercellular and intertissue signaling regulating oogenesis and ovulation.

## Supporting information

S1 Fig Replicate data set distribution, alignment for batch correction, follicular trajectory, and average expression across aligned clusters. (A) Scatter plot to show the relationship between total nGene and total nUMI for replicate 1 (REPL1: 7,053 cells) and replicate 2 (REPL2; 1,521 cells) post filtration. The plots show that the relationship is positively correlated (Pearson's correlation coefficient for REPL1: 0.87 and for REPL2: 0.88). P < 2.2*e*^−16^ for both plots. (B) UMAP plot containing cells from REPL1 and REPL2, aligned for batch correction using CCA. This plot shows 28 clusters of relevant cell types, comparable to Fig 1B. (C) Biweight mid-correlation (bicor) saturation plot for 30 CCV calculated to align the 2 replicates. The shared correlation between REPL1 and REPL2 show similar trends, and REPL2 shows lower correlation strength for the earliest CCV, which represent the most variable genes in the data set, thus showing how REPL1 is of higher quality. (D) UMAP plot to show the distribution of cells from the 2 replicates. (E) UMAP plot showing the distribution of only somatic/follicle-cell clusters from both replicates indicates cell fate trajectory. Follicle cells originate from the stem cell (FSC) cluster (indicated by the solid arrow) and assume polar and stalk cell fate (indicated by the dashed arrow). The remaining cells assume mitotic follicle-cell fate. This cluster subsequently splits into 2 distinct transcription states (solid arrow), representing cells in the Ant. and Post. egg chamber during follicular symmetry breaking. Some cells from resulting Ant. and Post. trajectories subsequently converge (dashed arrow) to form the migratory cells, whereas the Ant. and Post. trajectories terminally converge into the CL clusters. (F) Scatter plots to show correlation between REPL1 versus REPL2 average gene expression in each cluster belonging to the aligned UMAP shown in Fig S1B. Pearson's correlation coefficient values are listed for each plot, with *p* < 2.2*e*^−16^ for all plots. It is evident from these plots that REPL1 has a greater read depth that captures an increased number of feature counts. Also evident is the possible presence of multiplets in REPL2 that result in very high values of expression counts, possibly because of the less stringent filtering of REPL2 (because of a low cell number). Finally, REPL2 also shows reduced sampling of rarer cell types such as in hemocyte cluster, #22. Altogether these observations represent the challenges of incorporating replicate data sets in scRNA-Seq experiments. Raw sequence files available from SRA repository (SRX7814226). Processed files using Cell Ranger, Seurat, and Monocle are available through GEO database (GSE146040). Ant., anterior; CCV, canonical correlation vectors; CL, corpus luteum; CCA, canonical correlation analysis; FSC, follicle stem cell; nGene, number of genes; nUMI, number of unique molecular identifiers; Post., posterior; REPL1, replicate 1; REPL 2, replicate 2; scRNA-Seq, single-cell RNA sequencing; UMAP, Uniform Manifold Approximation Projection.
(TIF)

S2 Fig Strategy and validation of high-quality cell selection following doublet contamination processing. (A) Schematic representing the strategy of suspected doublet removal to obtain only high-quality cells in the final data set. The strategy is based on the idea that each individual cluster (representing cell type A, that is developmentally unrelated to cell type B) has its unique transcriptomic signature (yellow fragment within individual library captured in a droplet). Unique transcriptional signature of cell type B is represented by the red fragment. Doublets that may arise from accidental mixing of the 2 fragments are likely contaminants and have been removed from the data set after validation and careful examination of the genes. Individual clusters (or group of similar clusters) were selected and were cleaned for contaminating markers using this strategy to obtain high-quality cells. (B) Biweight midcorrelation (bicor) saturation plot for 50 CCV that were used to align the final and primary data sets. The 2 data sets are highly correlated even after stringent cleanup, indicating the fidelity of the final data set with that of the primary data set. (C) Violin plot to show the distribution of the canonical correlation projection vector (CC1) across the primary and the final data sets. (D) UMAP plot to show the distribution of cells from both the primary and final datasets that are aligned using CCA. (E) UMAP plot for the aligned data sets showing 31 clusters of cell types, comparable to that in Fig 1B. (F) Feature plots on the aligned UMAP, split by the 2 data sets, showing the expression of select markers used in doublet cleanup. The left column represents the cells used in the final data set, and the right column are the cells from the primary. The red color intensity represents gene expression relative to that across the data set. Raw sequence files available from SRA repository (SRX7814226). Processed files using Cell Ranger, Seurat, and Monocle are available through GEO database (GSE146040). CCV, canonical correlation vector; CCA, canonical correlation analysis; UMAP, Uniform Manifold Approximation Projection.
(TIF)

S3 Fig Data set preprocessing and relevant parameters. (A) Schematic for the scRNA-Seq analysis pipeline. (B) Violin plots for nGene, nUMI, and percent.mito for preprocessed data set (14,825 cells) and the final data set (7,053 cells). (C) Feature counts were log-normalized and scaled. Pre- and post-normalization plots are shown for total nUMIs and sum of gene expression counts. (D) Elbow Plot to show the ranking of the PCs based on the percentage of variance explained by each; 100 PCs have been computed for the final data set, 29 were selected for clustering, and 75 were selected for visualizing on the final UMAP shown in Fig 1B. Raw sequence files available from SRA repository (SRX7814226). Processed files using Cell Ranger, Seurat, and Monocle are available through GEO database (GSE146040). nGene, number of genes; nUMI, number of unique molecular identifiers; percent.mito, percentage of mitochondrial gene expression; PC, principal component; scRNA-Seq, single-cell RNA sequencing; UMAP, Uniform Manifold Approximation Projection.
(TIF)

S4 Fig Cluster resolution and ovarian and nonovarian cluster relationship information. (A) Clustering tree representing the relationship among all the clusters at resolutions 0.5 to 6.0. Example clustering shown for lowest (0.5) to highest (6) resolutions with cluster number ranging from 14 to 46. Cell-type identities were resolved by separating different clusters of transcriptional states and combining the ones that had no unique markers. (B) UMAP plot showing ovarian clusters (red), including somatic and germline cell types, and nonovarian clusters (blue), including cells from oviduct, muscle, hemocytes, and fat body. (C) UMAP plot showing the cell-cycle phase of all the cell clusters, based on the cell-cycle score assigned for genes in S2 File. (D) Plot showing the correlation between the different cell types. Clusters are numbered according to cell-type identities and numbers indicated in Fig 1B. Raw sequence files available from SRA repository (SRX7814226). Processed files using Cell Ranger, Seurat, and Monocle are available through GEO database (GSE146040). UMAP, Uniform Manifold Approximation Projection.
(TIF)

S1 File Known marker genes used to identify specific cell types. Table of marker genes used in this study to identify cell types with selected references.
(PDF)

S2 File Strategy used to separate dividing and nondividing cells. List of genes (adapted from Tirosh and colleagues [120]) used to assign “cell-cycle score” to each individual cell using genes for G2/M or S phase.
(XLSX)

S3 File Unique marker genes and statistics for each cell type. Differentially expressed genes and statistics for each cell type, as identified in Seurat (minimum expression in 25% cells of the cluster). Raw sequence files available from SRA repository (SRX7814226). Processed files using Cell Ranger, Seurat, and Monocle are available through GEO database (GSE146040).
(XLSX)

S4 File Markers used to select high-quality cells for specific clusters. Table of marker genes previously identified in different cell types with the associated references. Cells expressing 2 or more markers from different cell types were considered doublets and removed from the cluster (marked by X) to retain only high-quality cells.
(PDF)

## Abbreviations

Ant.: anterior
BC: border cell
CBC: cytoblast cell
CC: centripetal cell
CCA: canonical correlation analysis
CCV: Canonical Correlation vectors
CGM: correlated gene modules
CL: corpus luteum
DA: dorsal appendage
EBSS: Earle's Balanced Salt Solution
EGFR: epidermal growth factor receptor
FC: follicle cell
FSC: follicle stem cell
GFP: Green Fluorescent Protein
GO: gene ontology
GSC: germline stem cell
IGS: inner germarium sheath
JAK/STAT: Janus Kinase/Signal Transducer and Activator of Transcription
KEGG: Kyoto Encyclopedia of Genes and Genomes
M/E: mitosis-endocycle
MBFC: main body follicle cell
NADP: nicotinamide adenine dinucleotide phosphate
nGene: number of genes
nUMI: number of unique molecular identifiers
PBS: Phosphate-Buffered Saline
PBT: Phosphate-Buffered saline and Tween 20
Pc: polycomb
PC: principal component
PCA: principal component analysis
PFC: polar follicle cell
Post.: posterior
pre-FC: pre–follicle cell
REPL: replicate
RFP: Red Fluorescent Protein
RMCE: recombinase-mediated cassette exchange
SC: stretched cell
scRNA-seq: single-cell RNA sequencing
SNN: shared nearest neighbor
TF: terminal filament
TFC: terminal follicle cell
tSNE: t-Distributed Stochastic Neighbor Embedding
UMAP: Uniform Manifold Approximation Projection

## References

[1] Vied C, Reilein A, Field NS, Kalderon D. Regulation of stem cells by intersecting gradients of long-range niche signals. Dev Cell. 2012;23(4):836–848. 10.1016/j.devcel.2012.09.010 23079600 PMC3479678

[2] Xie T, Spradling AC. A niche maintaining germ line stem cells in the Drosophila ovary. Science. 2000;290(5490):328–330. 10.1126/science.290.5490.328 11030649

[3] Nystul T, Spradling A. An epithelial niche in the Drosophila ovary undergoes long-range stem cell replacement. Cell Stem Cell. 2007;1(3):277–285. 10.1016/j.stem.2007.07.009 18371362

[4] Losick VP, Morris LX, Fox DT, Spradling A. Drosophila stem cell niches: a decade of discovery suggests a unified view of stem cell regulation. Dev Cell. 2011;21(1):159–171. 10.1016/j.devcel.2011.06.018 21763616 PMC6894370

[5] Eliazer S, Buszczak M. Finding a niche: studies from the Drosophila ovary. Stem Cell Res Ther. 2011;2(6):45 10.1186/scrt86 22117545 PMC3340554

[6] Fadiga J, Nystul TG. The follicle epithelium in the Drosophila ovary is maintained by a small number of stem cells. Elife. 2019;8 10.7554/eLife.49050 31850843 PMC6946398

[7] Assa-Kunik E, Torres IL, Schejter ED, Johnston DS, Shilo BZ. Drosophila follicle cells are patterned by multiple levels of Notch signaling and antagonism between the Notch and JAK/STAT pathways. Development. 2007;134(6):1161–1169. 10.1242/dev.02800 17332535

[8] Klusza S, Deng WM. At the crossroads of differentiation and proliferation: Precise control of cell-cycle changes by multiple signaling pathways in Drosophila follicle cells. Bioessays. 2011;33(2):124–134. 10.1002/bies.201000089 21154780 PMC3891805

[9] Bosco G, Orr-Weaver TL. The cell cycle during oogenesis and early embryogenesis in Drosophila In: Advances in Developmental Biology and Biochemistry. vol. 12 of Gene Expression at the Beginning of Animal Development. Elsevier; 2002 p. 107–154.

[10] Cavaliere V, Donati A, Hsouna A, Hsu T, Gargiulo G. dAkt Kinase Controls Follicle Cell Size During Drosophila Oogenesis. Dev Dyn. 2005;232(3):845–854. 10.1002/dvdy.20333 15712201 PMC2265433

[11] Jia D, Tamori Y, Pyrowolakis G, Deng WM. Regulation of broad by the Notch pathway affects timing of follicle cell development. Dev Biol. 2014;392(1):52–61. 10.1016/j.ydbio.2014.04.024 24815210 PMC4296560

[12] Tootle TL, Williams D, Hubb A, Frederick R, Spradling A. Drosophila Eggshell Production: Identification of New Genes and Coordination by Pxt. PLoS ONE. 2011;6(5):e19943 10.1371/journal.pone.0019943 21637834 PMC3102670

[13] Wu X, Tanwar PS, Raftery LA. Drosophila follicle cells: morphogenesis in an eggshell. Semin Cell Dev Biol. 2008;19(3):271–282. 10.1016/j.semcdb.2008.01.004 18304845 PMC2430523

[14] Montell DJ, Rorth P, Spradling AC. slow border cells, a locus required for a developmentally regulated cell migration during oogenesis, encodes Drosophila C/EBP. Cell. 1992;71(1):51–62. 10.1016/0092-8674(92)90265-e 1394432

[15] Jang ACC, Chang YC, Bai J, Montell D. Border cell migration requires integration of spatial and temporal signals by the BTB protein Abrupt. Nat Cell Biol. 2009;11(5):569–579. 10.1038/ncb1863 19350016 PMC2675665

[16] Tamori Y, Deng WM. Tissue repair through cell competition and compensatory cellular hypertrophy in postmitotic epithelia. Dev Cell. 2013;25(4):350–363. 10.1016/j.devcel.2013.04.013 23685249 PMC3891806

[17] Tamori Y, Deng WM. Compensatory Cellular Hypertrophy: The Other Strategy for Tissue Homeostasis. Trends Cell Biol. 2014;24(4):230–237. 10.1016/j.tcb.2013.10.005 24239163 PMC4022146

[18] Jia D, Huang YC, Deng WM. Analysis of Cell Cycle Switches in Drosophila Oogenesis. Methods Mol Biol. 2015;1328:207–216. 10.1007/978-1-4939-2851-4_15 26324440 PMC5455776

[19] Deady LD, Shen W, Mosure SA, Spradling AC, Sun J. Matrix Metalloproteinase 2 Is Required for Ovulation and Corpus Luteum Formation in Drosophila. PLoS Genet. 2015;11(2):e1004989 10.1371/journal.pgen.1004989 25695427 PMC4335033

[20] Knapp EM, Li W, Sun J. Downregulation of homeodomain protein Cut is essential for Drosophila follicle maturation and ovulation. Development. 2019;146(18). 10.1242/dev.179002 31444217 PMC6765176

[21] Johnston DS. Using mutants, knockdowns, and transgenesis to investigate gene function in Drosophila. Wiley Interdisciplinary Reviews: Developmental Biology. 2013;2(5):587–613. 10.1002/wdev.101 24014449

[22] Venken KJT, Schulze KL, Haelterman NA, Pan H, He Y, Evans-Holm M, et al MiMIC: a highly versatile transposon insertion resource for engineering Drosophila melanogaster genes. Nat Methods. 2011;8(9):737–743. 10.1038/nmeth.1662 21985007 PMC3191940

[23] Buszczak M, Paterno S, Lighthouse D, Bachman J, Planck J, Owen S, et al The Carnegie Protein Trap Library: A Versatile Tool for Drosophila Developmental Studies. Genetics. 2007;175(3):1505–1531. 10.1534/genetics.106.065961 17194782 PMC1840051

[24] Lee PT, Zirin J, Kanca O, Lin WW, Schulze KL, Li-Kroeger D, et al A gene-specific T2A-GAL4 library for Drosophila. eLife;7 10.7554/eLife.35574 29565247 PMC5898912

[25] Diao F, Ironfield H, Luan H, Diao F, Shropshire W, Ewer J, et al Plug-and-Play Genetic Access to Drosophila Cell Types using Exchangeable Exon Cassettes. Cell Reports. 2015;10(8):1410–1421. 10.1016/j.celrep.2015.01.059 25732830 PMC4373654

[26] Zhang L, Ren F, Zhang Q, Chen Y, Wang B, Jiang J. The TEAD/TEF Family of Transcription Factor Scalloped Mediates Hippo Signaling in Organ Size Control. Developmental Cell. 2008;14(3):377–387. 10.1016/j.devcel.2008.01.006 18258485 PMC2292673

[27] Kolahi KS, White PF, Shreter DM, Classen AK, Bilder D, Mofrad MRK. Quantitative analysis of epithelial morphogenesis in Drosophila oogenesis: New insights based on morphometric analysis and mechanical modeling. Dev Biol. 2009;331(2):129–139. 10.1016/j.ydbio.2009.04.028 19409378 PMC3145632

[28] dos Santos G, Schroeder AJ, Goodman JL, Strelets VB, Crosby MA, Thurmond J, et al FlyBase: introduction of the Drosophila melanogaster Release 6 reference genome assembly and large-scale migration of genome annotations. Nucleic Acids Res. 2015;43(Database issue):D690–697. 10.1093/nar/gku1099 25398896 PMC4383921

[29] Butler A, Hoffman P, Smibert P, Papalexi E, Satija R. Integrating single-cell transcriptomic data across different conditions, technologies, and species. Nature Biotechnology. 2018;36(5):411–420. 10.1038/nbt.4096 29608179 PMC6700744

[30] Stuart T, Butler A, Hoffman P, Hafemeister C, Papalexi E, Mauck WM, et al Comprehensive Integration of Single-Cell Data. Cell. 2019;177(7):1888–1902.e21. 10.1016/j.cell.2019.05.031 31178118 PMC6687398

[31] Lun ATL, Riesenfeld S, Andrews T, Dao TP, Gomes T, Marioni JC, et al EmptyDrops: distinguishing cells from empty droplets in droplet-based single-cell RNA sequencing data. Genome Biology. 2019;20(1):63 10.1186/s13059-019-1662-y 30902100 PMC6431044

[32] Yang S, Corbett SE, Koga Y, Wang Z, Johnson WE, Yajima M, et al Decontamination of ambient RNA in single-cell RNA-seq with DecontX. bioRxiv. 2019; p. 704015. 10.1101/704015 PMC7059395 32138770

[33] DePasquale EAK, Schnell DJ, Van Camp PJ, Valiente-Alandí Í, Blaxall BC, Grimes HL, et al DoubletDecon: Deconvoluting Doublets from Single-Cell RNA-Sequencing Data. Cell Rep. 2019;29(6):1718–1727.e8. 10.1016/j.celrep.2019.09.082 31693907 PMC6983270

[34] Sanghavi P, Liu G, Veeranan-Karmegam R, Navarro C, Gonsalvez GB. Multiple Roles for Egalitarian in Polarization of the Drosophila Egg Chamber. Genetics. 2016;203(1):415–432. 10.1534/genetics.115.184622 27017624 PMC4858789

[35] Popodi E, Minoo P, Burke T, Waring GL. Organization and expression of a second chromosome follicle cell gene cluster in Drosophila. Dev Biol. 1988;127(2):248–256. 10.1016/0012-1606(88)90312-0 3132408

[36] Kim-Ha J, Smith JL, Macdonald PM. oskar mRNA is localized to the posterior pole of the Drosophila oocyte. Cell. 1991;66(1):23–35. 10.1016/0092-8674(91)90136-m 2070416

[37] Schonbaum CP, Lee S, Mahowald AP. The Drosophila yolkless gene encodes a vitellogenin receptor belonging to the low density lipoprotein receptor superfamily. PNAS. 1995;92(5):1485–1489. 10.1073/pnas.92.5.1485 7878005 PMC42544

[38] Schonbaum CP, Perrino JJ, Mahowald AP. Regulation of the vitellogenin receptor during Drosophila melanogaster oogenesis. Mol Biol Cell. 2000;11(2):511–521. 10.1091/mbc.11.2.511 10679010 PMC14789

[39] Noguereron MI, Mauzy-Melitz D, Waring GL. Drosophila dec-1 eggshell proteins are differentially distributed via a multistep extracellular processing and localization pathway. Dev Biol. 2000;225(2):459–470. 10.1006/dbio.2000.9805 10985863

[40] Fakhouri M, Elalayli M, Sherling D, Hall JD, Miller E, Sun X, et al Minor proteins and enzymes of the Drosophila eggshell matrix. Developmental Biology. 2006;293(1):127–141. 10.1016/j.ydbio.2006.01.028 16515779 PMC2701256

[41] Elalayli M, Hall JD, Fakhouri M, Neiswender H, Ellison TT, Han Z, et al Palisade is required in the Drosophila ovary for assembly and function of the protective vitelline membrane. Developmental Biology. 2008;319(2):359–369. 10.1016/j.ydbio.2008.04.035 18514182 PMC2536644

[42] Bernardi F, Cavaliere V, Andrenacci D, Gargiulo G. Dpp signaling down-regulates the expression of VM32E eggshell gene during Drosophila oogenesis. Developmental Dynamics. 2006;235(3):768–775. 10.1002/dvdy.20660 16372348

[43] Gargiulo G, Gigliotti S, Malva C, Graziani F. Cellular specificity of expression and regulation of Drosophila vitelline membrane protein 32E gene in the follicular epithelium: identification of cis-acting elements. Mechanisms of Development. 1991;35(3):193–203. 10.1016/0925-4773(91)90018-2 1768620

[44] Zhang Z, Stevens LM, Stein D. Sulfation of Eggshell Components by Pipe Defines Dorsal-Ventral Polarity in the Drosophila Embryo. Current Biology. 2009;19(14):1200–1205. 10.1016/j.cub.2009.05.050 19540119 PMC2733793

[45] Ayme-Southgate A, Lasko P, French C, Pardue ML. Characterization of the gene for mp20: a Drosophila muscle protein that is not found in asynchronous oscillatory flight muscle. J Cell Biol. 1989;108(2):521–531. 10.1083/jcb.108.2.521 2537318 PMC2115408

[46] Gunawan F, Arandjelovic M, Godt D. The Maf factor Traffic jam both enables and inhibits collective cell migration in Drosophila oogenesis. Development. 2013;140(13):2808–2817. 10.1242/dev.089896 23720044

[47] Okamoto N, Yamanaka N, Yagi Y, Nishida Y, Kataoka H, O'Connor MB, et al A Fat Body-Derived IGF-like Peptide Regulates Postfeeding Growth in Drosophila. Developmental Cell. 2009;17(6):885–891. 10.1016/j.devcel.2009.10.008 20059957 PMC4423396

[48] Bai H, Kang P, Tatar M. Drosophila insulin-like peptide-6 (dilp6) expression from fat body extends lifespan and represses secretion of Drosophila insulin-like peptide-2 from the brain. Aging Cell. 2012;11(6):978–985. 10.1111/acel.12000 22935001 PMC3500397

[49] Evans CJ, Liu T, Banerjee U. Drosophila hematopoiesis: markers and methods for molecular genetic analysis. Methods. 2014;68(1):242–251. 10.1016/j.ymeth.2014.02.038 24613936 PMC4051208

[50] Blondel VD, Guillaume JL, Lambiotte R, Lefebvre E. Fast unfolding of communities in large networks. J Stat Mech. 2008;2008(10):P10008 10.1088/1742-5468/2008/10/P10008

[51] Becht E, McInnes L, Healy J, Dutertre CA, Kwok IWH, Ng LG, et al Dimensionality reduction for visualizing single-cell data using UMAP. Nat Biotechnol. 2019;37(1):38–44. 10.1038/nbt.4314 30531897

[52] Zappia L, Oshlack A. Clustering trees: a visualization for evaluating clusterings at multiple resolutions. Gigascience. 2018;7(7). 10.1093/gigascience/giy083 30010766 PMC6057528

[53] Trapnell C, Cacchiarelli D, Grimsby J, Pokharel P, Li S, Morse M, et al Pseudo-temporal ordering of individual cells reveals dynamics and regulators of cell fate decisions. Nat Biotechnol. 2014;32(4):381–386. 24658644 10.1038/nbt.2859 PMC4122333

[54] Qiu X, Mao Q, Tang Y, Wang L, Chawla R, Pliner HA, et al Reversed graph embedding resolves complex single-cell trajectories. Nature Methods. 2017;14(10):979–982. 10.1038/nmeth.4402 28825705 PMC5764547

[55] Becker RA, Chambers JM, Wilks AR. The News Language: A Programming Environment for Data Analysis and Graphics. Pacific Grove, Calif: Chapman & Hall; 1988.

[56] Reimand J, Arak T, Adler P, Kolberg L, Reisberg S, Peterson H, et al g:Profiler-a web server for functional interpretation of gene lists (2016 update). Nucleic Acids Res. 2016;44(W1):W83–89. 10.1093/nar/gkw199 27098042 PMC4987867

[57] Mi H, Muruganujan A, Ebert D, Huang X, Thomas PD. PANTHER version 14: more genomes, a new PANTHER GO-slim and improvements in enrichment analysis tools. Nucleic Acids Res. 2019;47(Database issue):D419–D426. 10.1093/nar/gky1038 30407594 PMC6323939

[58] Rynes J, Donohoe CD, Frommolt P, Brodesser S, Jindra M, Uhlirova M. Activating Transcription Factor 3 Regulates Immune and Metabolic Homeostasis. Molecular and Cellular Biology. 2012;32(19):3949–3962. 10.1128/MCB.00429-12 22851689 PMC3457521

[59] Olswang-Kutz Y, Gertel Y, Benjamin S, Sela O, Pekar O, Arama E, et al Drosophila Past1 is involved in endocytosis and is required for germline development and survival of the adult fly. Journal of Cell Science. 2009;122(4):471–480. 10.1242/jcs.038521 19174465

[60] Casanueva MO, Ferguson EL. Germline stem cell number in the Drosophila ovary is regulated by redundant mechanisms that control Dpp signaling. Development. 2004;131(9):1881–1890. 10.1242/dev.01076 15105369

[61] Dansereau DA, Lasko P. The Development of Germline Stem Cells in Drosophila. Methods Mol Biol. 2008;450:3–26. 10.1007/978-1-60327-214-8_1 18370048 PMC2729445

[62] McKearin DM, Spradling AC. bag-of-marbles: a Drosophila gene required to initiate both male and female gametogenesis. Genes Dev. 1990;4(12B):2242–2251. 10.1101/gad.4.12b.2242 2279698

[63] Lantz V, Chang JS, Horabin JI, Bopp D, Schedl P. The Drosophila orb RNA-binding protein is required for the formation of the egg chamber and establishment of polarity. Genes & Development. 1994;8(5):598–613. 10.1101/gad.8.5.598 7523244

[64] Witt E, Benjamin S, Svetec N, Zhao L. Testis single-cell RNA-seq reveals the dynamics of de novo gene transcription and germline mutational bias in Drosophila. Elife. 2019;8 10.7554/eLife.47138 31418408 PMC6697446

[65] Tejedor F, Zhu XR, Kaltenbach E, Ackermann A, Baumann A, Canal I, et al minibrain: a new protein kinase family involved in postembryonic neurogenesis in Drosophila. Neuron. 1995;14(2):287–301. 10.1016/0896-6273(95)90286-4 7857639

[66] Park JW, Parisky K, Celotto AM, Reenan RA, Graveley BR. Identification of alternative splicing regulators by RNA interference in Drosophila. PNAS. 2004;101(45):15974–15979. 10.1073/pnas.0407004101 15492211 PMC528766

[67] Margolis J, Spradling A. Identification and behavior of epithelial stem cells in the Drosophila ovary. Development. 1995;121(11):3797–3807. 8582289 10.1242/dev.121.11.3797

[68] Shyu LF, Sun J, Chung HM, Huang YC, Deng WM. Notch signaling and developmental cell-cycle arrest in Drosophila polar follicle cells. Mol Biol Cell. 2009;20(24):5064–5073. 10.1091/mbc.E09-01-0004 19846665 PMC2793284

[69] Tworoger M, Larkin MK, Bryant Z, Ruohola-Baker H. Mosaic analysis in the drosophila ovary reveals a common hedgehog-inducible precursor stage for stalk and polar cells. Genetics. 1999;151(2):739–748. 9927465 10.1093/genetics/151.2.739 PMC1460513

[70] Chang YC, Jang ACC, Lin CH, Montell DJ. Castor is required for Hedgehog-dependent cell-fate specification and follicle stem cell maintenance in Drosophila oogenesis. PNAS. 2013;110(19):E1734–E1742. 10.1073/pnas.1300725110 23610413 PMC3651482

[71] Gaudet P, Livstone MS, Lewis SE, Thomas PD. Phylogenetic-based propagation of functional annotations within the Gene Ontology consortium. Brief Bioinform. 2011;12(5):449–462. 10.1093/bib/bbr042 21873635 PMC3178059

[72] Guruharsha KG, Rual JF, Zhai B, Mintseris J, Vaidya P, Vaidya N, et al A protein complex network of Drosophila melanogaster. Cell. 2011;147(3):690–703. 10.1016/j.cell.2011.08.047 22036573 PMC3319048

[73] Banerjee S, Blauth K, Peters K, Rogers SL, Fanning AS, Bhat MA. Drosophila Neurexin IV Interacts with Roundabout and Is Required for Repulsive Midline Axon Guidance. J Neurosci. 2010;30(16):5653–5667. 10.1523/JNEUROSCI.6187-09.2010 20410118 PMC2869042

[74] Wu MF, Liao CY, Wang LY, Chang JT. The role of Slit-Robo signaling in the regulation of tissue barriers. Tissue Barriers. 2017;5(2). 10.1080/21688370.2017.1331155 28598714 PMC5501134

[75] Grammont M, Irvine KD. Organizer activity of the polar cells during Drosophila oogenesis. Development. 2002;129(22):5131–5140. 12399305 10.1242/dev.129.22.5131

[76] Arndt V, Dick N, Tawo R, Dreiseidler M, Wenzel D, Hesse M, et al Chaperone-assisted selective autophagy is essential for muscle maintenance. Curr Biol. 2010;20(2):143–148. 10.1016/j.cub.2009.11.022 20060297

[77] Deng WM, Althauser C, Ruohola-Baker H. Notch-Delta signaling induces a transition from mitotic cell cycle to endocycle in Drosophila follicle cells. Development. 2001;128(23):4737–4746. 11731454 10.1242/dev.128.23.4737

[78] Sun J, Deng WM. Notch-dependent downregulation of the homeodomain gene cut is required for the mitotic cycle/endocycle switch and cell differentiation in Drosophila follicle cells. Development. 2005;132(19):4299–4308. 10.1242/dev.02015 16141223 PMC3891799

[79] Sun J, Deng WM. Hindsight mediates the role of notch in suppressing hedgehog signaling and cell proliferation. Dev Cell. 2007;12(3):431–442. 10.1016/j.devcel.2007.02.003 17336908 PMC1851662

[80] Kankel MW, Hurlbut GD, Upadhyay G, Yajnik V, Yedvobnick B, Artavanis-Tsakonas S. Investigating the Genetic Circuitry of Mastermind in Drosophila, a Notch Signal Effector. Genetics. 2007;177(4):2493–2505. 10.1534/genetics.107.080994 18073442 PMC2219471

[81] Horne-Badovinac S. The Drosophila Egg Chamber—A New Spin on How Tissues Elongate. Integr Comp Biol. 2014;54(4):667–676. 10.1093/icb/icu067 24920751 PMC4229889

[82] Jordan KC, Clegg NJ, Blasi JA, Morimoto AM, Sen J, Stein D, et al The homeobox gene mirror links EGF signalling to embryonic dorso-ventral axis formation through Notch activation. Nat Genet. 2000;24(4):429–433. 10.1038/74294 10742112

[83] D'Alterio C, Tran DDD, Yeung MWYA, Hwang MSH, Li MA, Arana CJ, et al Drosophila melanogaster Cad99C, the orthologue of human Usher cadherin PCDH15, regulates the length of microvilli. J Cell Biol. 2005;171(3):549–558. 10.1083/jcb.200507072 16260500 PMC2171266

[84] Murray MJ, Davidson CM, Hayward NM, Brand AH. The Fes/Fer non-receptor tyrosine kinase cooperates with Src42A to regulate dorsal closure in Drosophila. Development. 2006;133(16):3063–3073. 10.1242/dev.02467 16831834

[85] Martin D, Zusman S, Li X, Williams EL, Khare N, DaRocha S, et al wing blister, a new Drosophila laminin alpha chain required for cell adhesion and migration during embryonic and imaginal development. J Cell Biol. 1999;145(1):191–201. 10.1083/jcb.145.1.191 10189378 PMC2148222

[86] Du J, Zhang J, He T, Li Y, Su Y, Tie F, et al Stuxnet Facilitates the Degradation of Polycomb Protein during Development. Developmental Cell. 2016;37(6):507–519. 10.1016/j.devcel.2016.05.013 27326929 PMC7365346

[87] Deng W, Leaper K, Bownes M. A targeted gene silencing technique shows that Drosophila myosin VI is required for egg chamber and imaginal disc morphogenesis. J Cell Sci. 1999;112(21):3677–3690. 10523504 10.1242/jcs.112.21.3677

[88] Geisbrecht ER, Montell DJ. Myosin VI is required for E-cadherin-mediated border cell migration. Nat Cell Biol. 2002;4(8):616–620. 10.1038/ncb830 12134162

[89] Sahu A, Ghosh R, Deshpande G, Prasad M. A Gap Junction Protein, Inx2, Modulates Calcium Flux to Specify Border Cell Fate during Drosophila oogenesis. PLoS Genet. 2017;13(1):e1006542 10.1371/journal.pgen.1006542 28114410 PMC5256874

[90] Hill E, Broadbent ID, Chothia C, Pettitt J. Cadherin superfamily proteins in Caenorhabditis elegans and Drosophila melanogaster11Edited by G. von Heijne. Journal of Molecular Biology. 2001;305(5):1011–1024. 10.1006/jmbi.2000.4361 11162110

[91] Glowinski C, Liu RHS, Chen X, Darabie A, Godt D. Myosin VIIA regulates microvillus morphogenesis and interacts with cadherin Cad99C in Drosophila oogenesis. J Cell Sci. 2014;127(22):4821–4832. 10.1242/jcs.099242 25236597

[92] Carney GE, Bowen NJ. p24 proteins, intracellular trafficking, and behavior: Drosophila melanogaster provides insights and opportunities. Biol Cell. 2004;96(4):271–278. 10.1016/j.biolcel.2004.01.004 15145531

[93] Simoes S, Denholm B, Azevedo D, Sotillos S, Martin P, Skaer H, et al Compartmentalisation of Rho regulators directs cell invagination during tissue morphogenesis. Development. 2006;133(21):4257–4267. 10.1242/dev.02588 17021037

[94] Timmons AK, Mondragon AA, Meehan TL, McCall K. Control of non-apoptotic nurse cell death by engulfment genes in Drosophila. Fly (Austin). 2016;11(2):104–111. 10.1080/19336934.2016.1238993 27686122 PMC5406165

[95] Meehan T, Joudi TF, Lord A, Taylor JD, Habib CS, Peterson J, et al Components of the Engulfment Machinery Have Distinct Roles in Corpse Processing. PLoS ONE. 2016;11:e0158217 10.1371/journal.pone.0158217 27347682 PMC4922577

[96] Tryselius Y, Hultmark D. Cysteine proteinase 1 (CP1), a cathepsin L-like enzyme expressed in the Drosophila melanogaster haemocyte cell line mbn-2. Insect Molecular Biology. 1997;6(2):173–181. 10.1111/j.1365-2583.1997.tb00085.x 9099581

[97] Kongton K, McCall K, Phongdara A. Identification of gamma-interferon-inducible lysosomal thiol reductase (GILT) homologues in the fruit fly Drosophila melanogaster. Developmental & Comparative Immunology. 2014;44(2):389–396. 10.1016/j.dci.2014.01.007 24491521

[98] Sellin J, Schulze H, Paradis M, Gosejacob D, Papan C, Shevchenko A, et al Characterization of Drosophila Saposin-related mutants as a model for lysosomal sphingolipid storage diseases. Disease Models & Mechanisms. 2017;10(6):737–750. 10.1242/dmm.027953 28389479 PMC5483003

[99] Gregory CD, Devitt A. The macrophage and the apoptotic cell: an innate immune interaction viewed simplistically? Immunology. 2004;113(1):1–14. 10.1111/j.1365-2567.2004.01959.x 15312130 PMC1782541

[100] Emelyanov AV, Rabbani J, Mehta M, Vershilova E, Keogh MC, Fyodorov DV. Drosophila TAP/p32 is a core histone chaperone that cooperates with NAP-1, NLP, and nucleophosmin in sperm chromatin remodeling during fertilization. Genes Dev. 2014;28(18):2027–2040. 10.1101/gad.248583.114 25228646 PMC4173154

[101] Deady LD, Li W, Sun J. The zinc-finger transcription factor Hindsight regulates ovulation competency of Drosophila follicles. Elife. 2017;6 10.7554/eLife.29887 29256860 PMC5768419

[102] Colombani J, Andersen DS, Leopold P. Secreted Peptide Dilp8 Coordinates Drosophila Tissue Growth with Developmental Timing. Science. 2012;336(6081):582–585. 10.1126/science.1216689 22556251

[103] Leulier F, Ribeiro PS, Palmer E, Tenev T, Takahashi K, Robertson D, et al Systematic in vivo RNAi analysis of putative components of the Drosophila cell death machinery. Cell Death & Differentiation. 2006;13(10):1663 10.1038/sj.cdd.4401868 16485033

[104] Yazdani U, Huang Z, Terman JR. The glucose transporter (GLUT4) enhancer factor is required for normal wing positioning in Drosophila. Genetics. 2008;178(2):919–929. 10.1534/genetics.107.078030 18245850 PMC2248331

[105] Rylett CM, Walker MJ, Howell GJ, Shirras AD, Isaac RE. Male accessory glands of Drosophila melanogaster make a secreted angiotensin I-converting enzyme (ANCE), suggesting a role for the peptide-processing enzyme in seminal fluid. Journal of Experimental Biology. 2007;210(20):3601–3606. 10.1242/jeb.009035 17921161

[106] Dostert C, Jouanguy E, Irving P, Troxler L, Galiana-Arnoux D, Hetru C, et al The Jak-STAT signaling pathway is required but not sufficient for the antiviral response of drosophila. Nat Immunol. 2005;6(9):946–953. 10.1038/ni1237 16086017

[107] Li W, Young JF, Sun J. NADPH oxidase-generated reactive oxygen species in mature follicles are essential for Drosophila ovulation. Proc Natl Acad Sci USA. 2018;115(30):7765–7770. 10.1073/pnas.1800115115 29987037 PMC6065002

[108] Ritsick DR, Edens WA, Finnerty V, Lambeth JD. Nox regulation of smooth muscle contraction. Free Radic Biol Med. 2007;43(1):31–38. 10.1016/j.freeradbiomed.2007.03.006 17561091 PMC1989158

[109] Lim J, Sabandal PR, Fernandez A, Sabandal JM, Lee HG, Evans P, et al The Octopamine Receptor Octβ2R Regulates Ovulation in Drosophila melanogaster. PLoS ONE. 2014;9(8):e104441 10.1371/journal.pone.0104441 25099506 PMC4123956

[110] Kemp C, Imler JL. Antiviral immunity in drosophila. Curr Opin Immunol. 2009;21(1):3–9. 10.1016/j.coi.2009.01.007 19223163 PMC2709802

[111] Adams EC, Hertig AT. STUDIES ON THE HUMAN CORPUS LUTEUM: I. Observations on the Ultrastructure of Development and Regression of the Luteal Cells During the Menstrual Cycle. The Journal of Cell Biology. 1969;41(3):696–715. 10.1083/jcb.41.3.696 5768870 PMC2107822

[112] Niswender GD, Juengel JL, Silva PJ, Rollyson MK, McIntush EW. Mechanisms Controlling the Function and Life Span of the Corpus Luteum. Physiological Reviews. 2000;80(1):1–29. 10.1152/physrev.2000.80.1.1 10617764

[113] Care AS, Diener KR, Jasper MJ, Brown HM, Ingman WV, Robertson SA. Macrophages regulate corpus luteum development during embryo implantation in mice. J Clin Invest. 2013;123(8):3472–3487. 10.1172/JCI60561 23867505 PMC3726148

[114] Wu R, Van der Hoek KH, Ryan NK, Norman RJ, Robker RL. Macrophage contributions to ovarian function. Hum Reprod Update. 2004;10(2):119–133. 10.1093/humupd/dmh011 15073142

[115] Chapman AR, Lee DF, Cai W, Ma W, Li X, Sun W, et al Correlated Gene Modules Uncovered by Single-Cell Transcriptomics with High Detectability and Accuracy. bioRxiv. 2020; p. 2019.12.31.892190. 10.1101/2019.12.31.892190

[116] Hwang B, Lee JH, Bang D. Single-cell RNA sequencing technologies and bioinformatics pipelines. Exp Mol Med. 2018;50(8):96 10.1038/s12276-018-0071-8 30089861 PMC6082860

[117] Luecken MD, Theis FJ. Current best practices in single-cell RNA-seq analysis: a tutorial. Molecular Systems Biology. 2019;15(6):e8746 10.15252/msb.20188746 31217225 PMC6582955

[118] Rust K, Byrnes L, Yu KS, Park JS, Sneddon JB, Tward AD, et al A Single-Cell Atlas and Lineage Analysis of the Adult Drosophila Ovary. bioRxiv. 2019; p. 798223. 10.1101/798223 PMC7648648 33159074

[119] Slaidina M, Banisch TU, Gupta S, Lehmann R. A single-cell atlas of the developing Drosophila ovary identifies follicle stem cell progenitors. Genes Dev. 2020; 10.1101/gad.330464.119 31919193 PMC7000915

[120] Tirosh I, Izar B, Prakadan SM, Wadsworth MH, Treacy D, Trombetta JJ, et al Dissecting the multicellular ecosystem of metastatic melanoma by single-cell RNA-seq. Science. 2016;352(6282):189–196. 10.1126/science.aad0501 27124452 PMC4944528

