# Supplementary figures and images for: A single-cell atlas of adult Drosophila ovary identifies transcriptional programs and somatic cell lineage regulating oogenesis
Source: PLoS Biol. 2020 Apr 27;18(4):e3000538. doi: 10.1371/journal.pbio.3000538 (PMC7205450; doi:10.1371/journal.pbio.3000538)

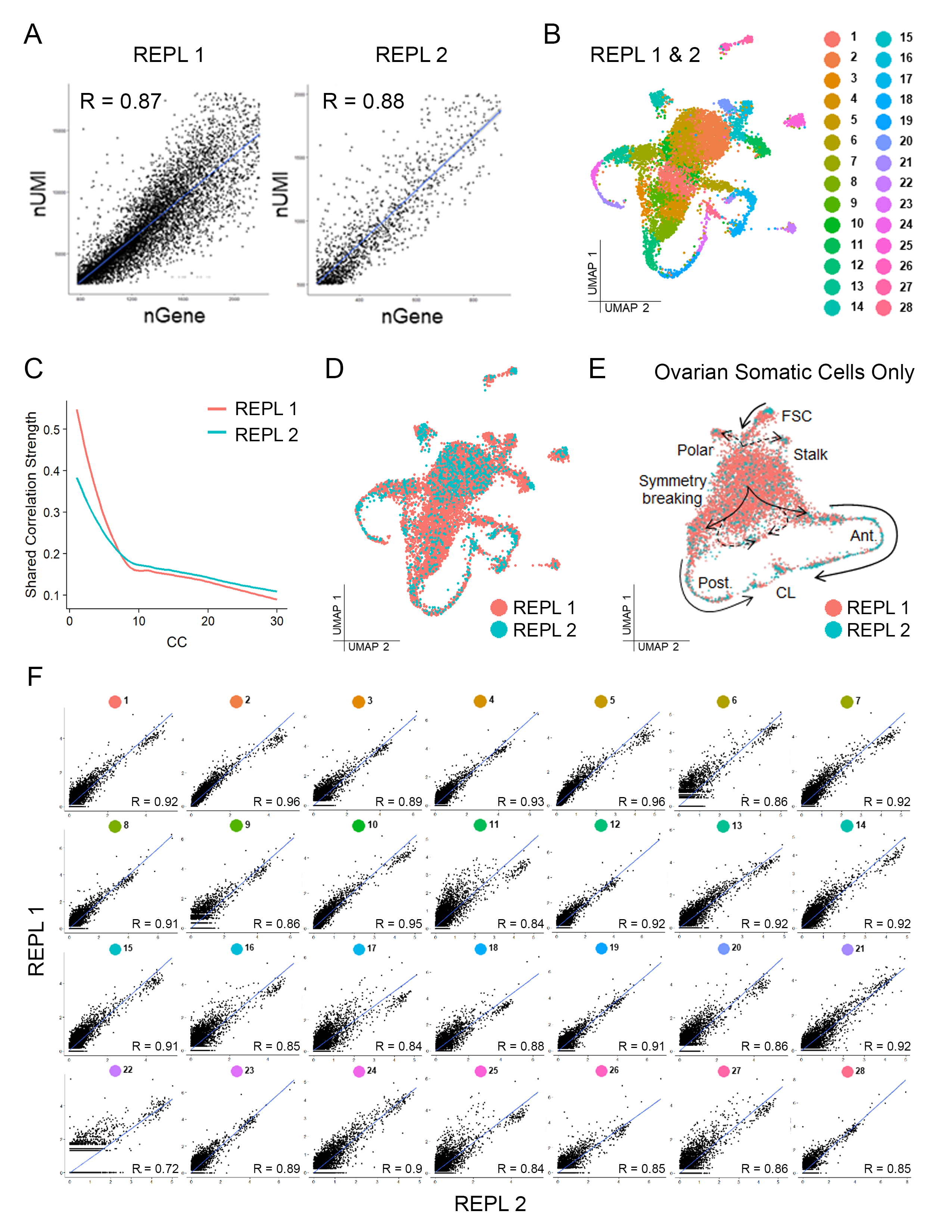

Supplement: S1 Fig — (A) Scatter plot to show the relationship between total nGene and total nUMI for replicate 1 (REPL1: 7,053 cells) and replicate 2 (REPL2; 1,521 cells) post filtration. The plots show that the relationship is positively correlated (Pearson's correlation coefficient for REPL1: 0.87 and for REPL2: 0.88). P < 2.2e−16 for both plots. (B) UMAP plot containing cells from REPL1 and REPL2, aligned for batch correction using CCA. This plot shows 28 clusters of relevant cell types, comparable to Fig 1B. (C) Biweight mid-correlation (bicor) saturation plot for 30 CCV calculated to align the 2 replicates. The shared correlation between REPL1 and REPL2 show similar trends, and REPL2 shows lower correlation strength for the earliest CCV, which represent the most variable genes in the data set, thus showing how REPL1 is of higher quality. (D) UMAP plot to show the distribution of cells from the 2 replicates. (E) UMAP plot showing the distribution of only somatic/follicle-cell clusters from both replicates indicates cell fate trajectory. Follicle cells originate from the stem cell (FSC) cluster (indicated by the solid arrow) and assume polar and stalk cell fate (indicated by the dashed arrow). The remaining cells assume mitotic follicle-cell fate. This cluster subsequently splits into 2 distinct transcription states (solid arrow), representing cells in the Ant. and Post. egg chamber during follicular symmetry breaking. Some cells from resulting Ant. and Post. trajectories subsequently converge (dashed arrow) to form the migratory cells, whereas the Ant. and Post. trajectories terminally converge into the CL clusters. (F) Scatter plots to show correlation between REPL1 versus REPL2 average gene expression in each cluster belonging to the aligned UMAP shown in Fig S1B. Pearson's correlation coefficient values are listed for each plot, with p < 2.2e−16 for all plots. It is evident from these plots that REPL1 has a greater read depth that captures an increased number of feature counts. [file pbio.3000538.s001.tif]

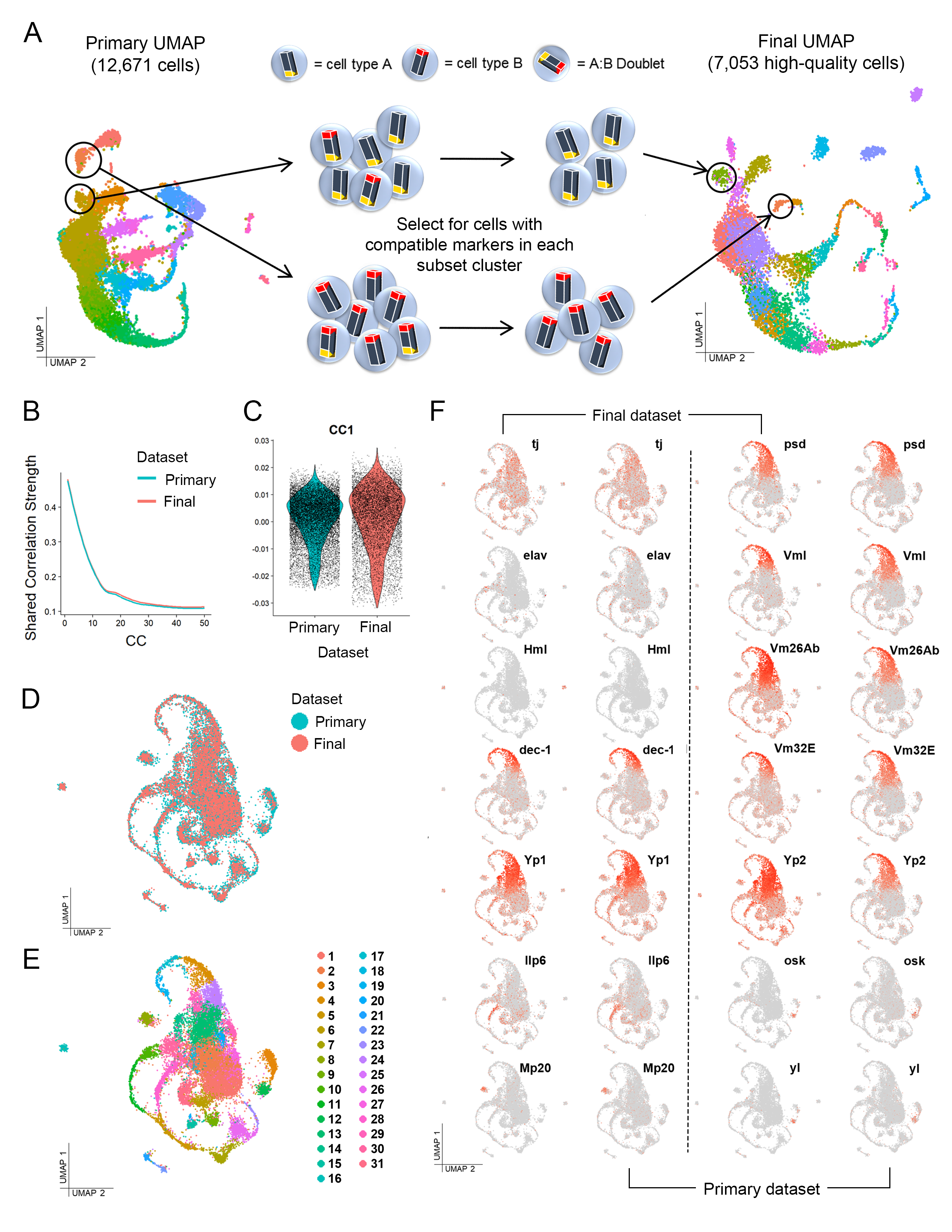

Supplement: S2 Fig — (A) Schematic representing the strategy of suspected doublet removal to obtain only high-quality cells in the final data set. The strategy is based on the idea that each individual cluster (representing cell type A, that is developmentally unrelated to cell type B) has its unique transcriptomic signature (yellow fragment within individual library captured in a droplet). Unique transcriptional signature of cell type B is represented by the red fragment. Doublets that may arise from accidental mixing of the 2 fragments are likely contaminants and have been removed from the data set after validation and careful examination of the genes. Individual clusters (or group of similar clusters) were selected and were cleaned for contaminating markers using this strategy to obtain high-quality cells. (B) Biweight midcorrelation (bicor) saturation plot for 50 CCV that were used to align the final and primary data sets. The 2 data sets are highly correlated even after stringent cleanup, indicating the fidelity of the final data set with that of the primary data set. (C) Violin plot to show the distribution of the canonical correlation projection vector (CC1) across the primary and the final data sets. (D) UMAP plot to show the distribution of cells from both the primary and final datasets that are aligned using CCA. (E) UMAP plot for the aligned data sets showing 31 clusters of cell types, comparable to that in Fig 1B. (F) Feature plots on the aligned UMAP, split by the 2 data sets, showing the expression of select markers used in doublet cleanup. The left column represents the cells used in the final data set, and the right column are the cells from the primary. The red color intensity represents gene expression relative to that across the data set. Raw sequence files available from SRA repository (SRX7814226). Processed files using Cell Ranger, Seurat, and Monocle are available through GEO database (GSE146040). CCV, canonical correlation vector; CCA, canonical correlation analy [file pbio.3000538.s002.tif]

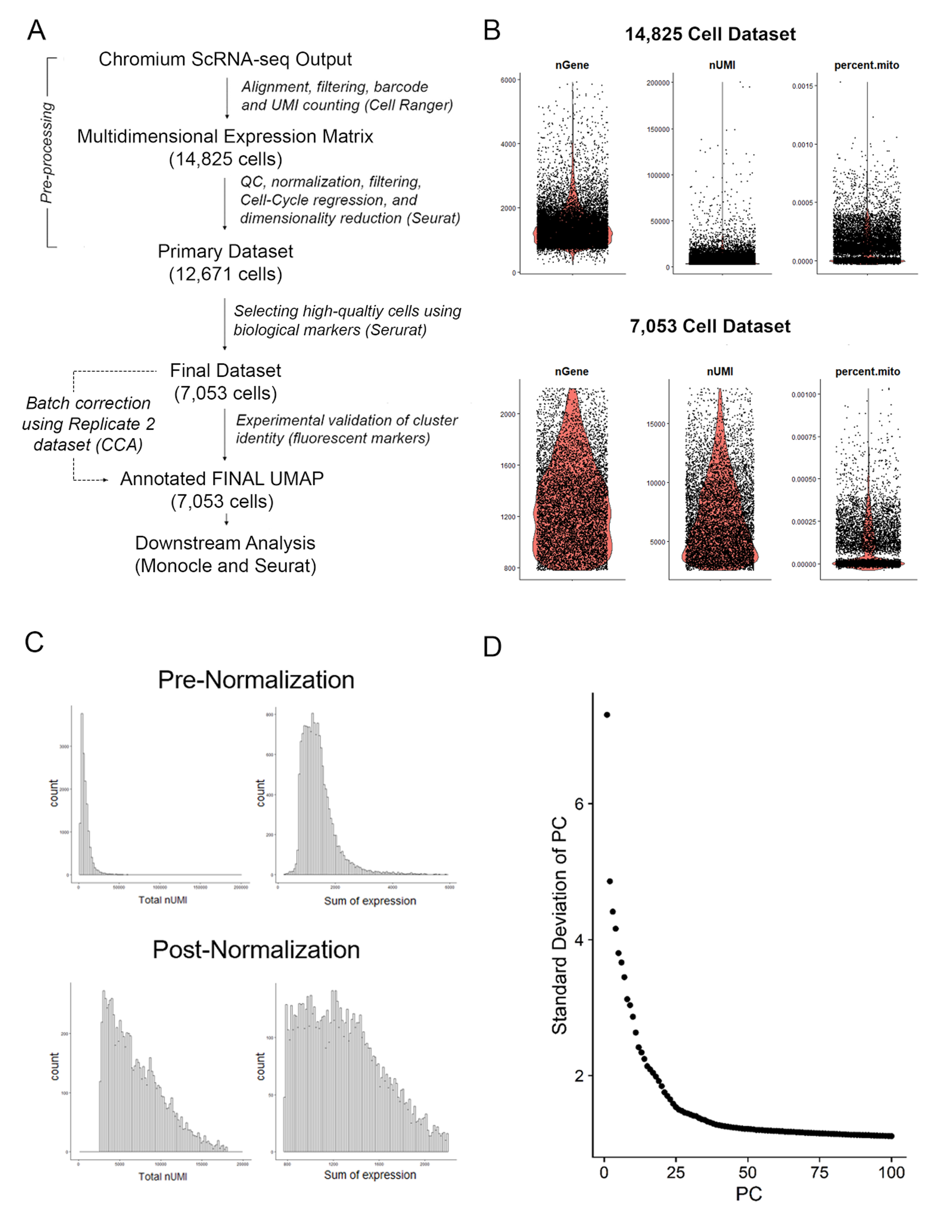

Supplement: S3 Fig — (A) Schematic for the scRNA-Seq analysis pipeline. (B) Violin plots for nGene, nUMI, and percent.mito for preprocessed data set (14,825 cells) and the final data set (7,053 cells). (C) Feature counts were log-normalized and scaled. Pre- and post-normalization plots are shown for total nUMIs and sum of gene expression counts. (D) Elbow Plot to show the ranking of the PCs based on the percentage of variance explained by each; 100 PCs have been computed for the final data set, 29 were selected for clustering, and 75 were selected for visualizing on the final UMAP shown in Fig 1B. Raw sequence files available from SRA repository (SRX7814226). Processed files using Cell Ranger, Seurat, and Monocle are available through GEO database (GSE146040). nGene, number of genes; nUMI, number of unique molecular identifiers; percent.mito, percentage of mitochondrial gene expression; PC, principal component; scRNA-Seq, single-cell RNA sequencing; UMAP, Uniform Manifold Approximation Projection. (TIF) [file pbio.3000538.s003.tif]

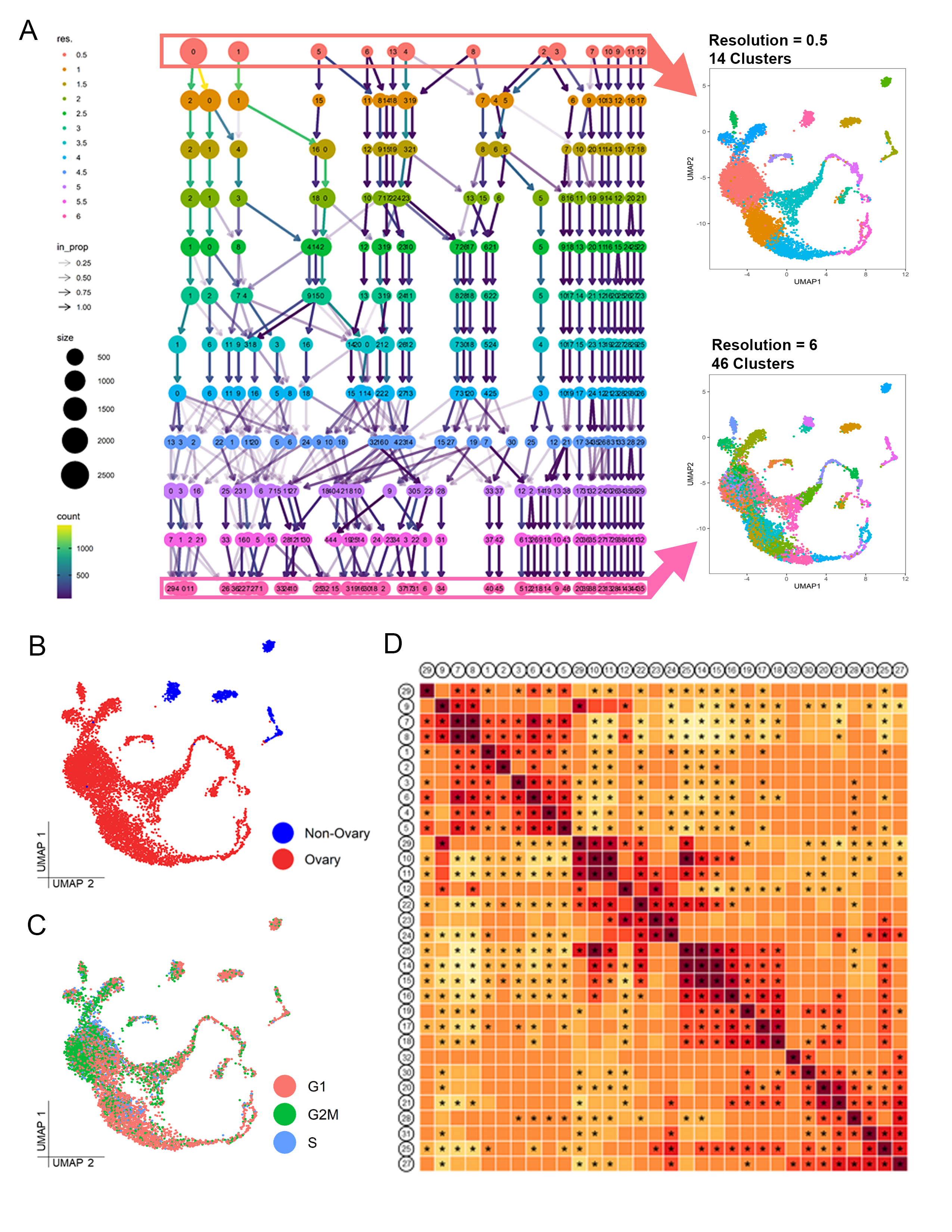

Supplement: S4 Fig — (A) Clustering tree representing the relationship among all the clusters at resolutions 0.5 to 6.0. Example clustering shown for lowest (0.5) to highest (6) resolutions with cluster number ranging from 14 to 46. Cell-type identities were resolved by separating different clusters of transcriptional states and combining the ones that had no unique markers. (B) UMAP plot showing ovarian clusters (red), including somatic and germline cell types, and nonovarian clusters (blue), including cells from oviduct, muscle, hemocytes, and fat body. (C) UMAP plot showing the cell-cycle phase of all the cell clusters, based on the cell-cycle score assigned for genes in S2 File. (D) Plot showing the correlation between the different cell types. Clusters are numbered according to cell-type identities and numbers indicated in Fig 1B. Raw sequence files available from SRA repository (SRX7814226). Processed files using Cell Ranger, Seurat, and Monocle are available through GEO database (GSE146040). UMAP, Uniform Manifold Approximation Projection. (TIF) [file pbio.3000538.s004.tif]
