# Supplementary material for: A single-cell atlas of adult Drosophila ovary identifies transcriptional programs and somatic cell lineage regulating oogenesis
Source: PLoS Biol. 2020 Apr 27;18(4):e3000538. doi: 10.1371/journal.pbio.3000538 (PMC7205450; doi:10.1371/journal.pbio.3000538)
Supplement: S1 File — Table of marker genes used in this study to identify cell types with selected references. (PDF) [file pbio.3000538.s005.pdf]

Markers	Cell Types	References
vas, bam	Germline stem cells Cystoblast	[1–3]
orb	Germline cells from region 2 of the germarium onward	[4]
Wnt4	Somatic cells in the germarium: escort cells, follicle stem cells, cap cells, terminal filament cells	[4,5]
upd1	Polar cells	[6]
ct	Stage 2-6: follicle cells (mitotic)	[7]
psd	Stage 8-14: follicle cells	[8]
Vml	Stage 10: follicle cells	[9]
dec-1	Stage 9-12: follicle cells	[10,11]
ttk	Stage 8-10B: follicle cells	[12,13]
Fcp3C	Stage 10B: follicle cells	[10,14]
Cad86C	Stage 10B: dorsal appendage forming cells (roof cells)<br>Stage 11-12: All dorsal columnar follicle cells	[15]
Cad74A	Stage 10B: main body follicle cells and posterior cells but not roof cells	[15]
yellow-g	Stage 12: follicle cells	[10]
Femcoat	Stage 14: follicle cells	[10,16]
mirr	Somatic cells in the germarium Stage 6: centripetal and main body follicle cell, Stage 10: dorsal anterior main body follicle cells and centripetal cells	[17]
Cad99C	Stage 2-5: terminal follicle cells. Stage 6-7: posterior follicle cells (contacting oocyte)<br>Stage 10A: all main body follicle cells, centripetal cells, and posterior follicle cells<br>Stage 10B: centripetal cells	[18]
slbo	Stage 9-14: border cells, centripetal cells, and posterior cells	[19]
peb	Stage 7-10: follicle cells Stage 14: follicle cells Corpus luteum cells	[20,21]
Mmp2	Stage 14: anterior and posterior follicle cells<br>Corpus luteum: anterior and posterior cells	[22]
Mp20	Muscle sheath cells	[23]
Ilp6	Adipocytes	[24]
Hml	Hemocytes	[25]

References:

1. Hay B, Jan LY, Jan YN. A protein component of Drosophila polar granules is encoded by vasa and has extensive sequence similarity to ATP-dependent helicases. Cell. 1988;55: 577–587. doi:10.1016/0092-8674(88)90216-4

2. McKearin DM, Spradling AC. bag-of-marbles: a Drosophila gene required to initiate both male and female gametogenesis. Genes Dev. 1990;4: 2242–2251. doi:10.1101/gad.4.12b.2242

3. Lasko PF, Ashburner M. The product of the Drosophila gene vasa is very similar to eukaryotic initiation factor-4A. Nature. 1988;335: 611–617. doi:10.1038/335611a0

4. Cohen ED, Mariol M-C, Wallace RMH, Weyers J, Kamberov YG, Pradel J, et al. DWnt4 Regulates Cell Movement and Focal Adhesion Kinase during Drosophila Ovarian Morphogenesis. Developmental Cell. 2002;2: 437–448. doi:10.1016/S1534-5807(02)00142-9

5. Liu Z, Zhong G, Chai PC, Luo L, Liu S, Yang Y, et al. Coordinated niche-associated signals promote germline homeostasis in the Drosophila ovary. J Cell Biol. 2015;211: 469–484. doi:10.1083/jcb.201503033

6. Assa-Kunik E, Torres IL, Schejter ED, Johnston DS, Shilo B-Z. Drosophila follicle cells are patterned by multiple levels of Notch signaling and antagonism between the Notch and JAK/STAT pathways. Development. 2007;134: 1161–1169. doi:10.1242/dev.02800

7. Sun J, Deng W-M. Notch-dependent downregulation of the homeodomain gene cut is required for the mitotic cycle/endocycle switch and cell differentiation in Drosophila follicle cells. Development. 2005;132: 4299–4308. doi:10.1242/dev.02015

8. Elalayli M, Hall JD, Fakhouri M, Neiswender H, Ellison TT, Han Z, et al. Palisade is required in the Drosophila ovary for assembly and function of the protective vitelline membrane. Developmental Biology. 2008;319: 359–369. doi:10.1016/j.ydbio.2008.04.035

9. Zhang Z, Stevens LM, Stein D. Sulfation of Eggshell Components by Pipe Defines Dorsal-Ventral Polarity in the Drosophila Embryo. Current Biology. 2009;19: 1200–1205. doi:10.1016/j.cub.2009.05.050

10. Tootle TL, Williams D, Hubb A, Frederick R, Spradling A. Drosophila Eggshell Production: Identification of New Genes and Coordination by Pxt. PLOS ONE. 2011;6: e19943. doi:10.1371/journal.pone.0019943

11. Noguerón MI, Mauzy-Melitz D, Waring GL. Drosophila dec-1 eggshell proteins are differentially distributed via a multistep extracellular processing and localization pathway. Dev Biol. 2000;225: 459–470. doi:10.1006/dbio.2000.9805

12. Sun J, Smith L, Armento A, Deng W-M. Regulation of the endocycle/gene amplification switch by Notch and ecdysone signaling. J Cell Biol. 2008;182: 885–896. doi:10.1083/jcb.200802084

13. Boyle MJ, Berg CA. Control in time and space: Tramtrack69 cooperates with Notch and Ecdysone to repress ectopic fate and shape changes during Drosophila egg chamber maturation. Development. 2009;136: 4187–4197. doi:10.1242/dev.042770

14. Burke T, Waring GL, Popodi E, Minoo P. Characterization and sequence of follicle cell genes selectively expressed during vitelline membrane formation in Drosophila. Developmental Biology. 1987;124: 441–450. doi:10.1016/0012-1606(87)90497-0

15. Zartman JJ, Kanodia JS, Yakoby N, Schafer X, Watson C, Schlichting K, et al. Expression patterns of cadherin genes in Drosophila oogenesis. Gene Expr Patterns. 2009;9: 31–36. doi:10.1016/j.gep.2008.09.001

16. Kim C, Han K, Kim J, Yi JS, Kim C, Yim J, et al. Femcoat, a novel eggshell protein in Drosophila: functional analysis by double stranded RNA interference. Mechanisms of Development. 2002;110: 61–70. doi:10.1016/S0925-4773(01)00559-7

17. Jordan KC, Clegg NJ, Blasi JA, Morimoto AM, Sen J, Stein D, et al. The homeobox gene mirror links EGF signalling to embryonic dorso-ventral axis formation through Notch activation. Nat Genet. 2000;24: 429–433. doi:10.1038/74294

18. D'Alterio C, Tran DDD, Yeung MWYA, Hwang MSH, Li MA, Arana CJ, et al. Drosophila melanogaster Cad99C, the orthologue of human Usher cadherin PCDH15, regulates the length of microvilli. J Cell Biol. 2005;171: 549–558. doi:10.1083/jcb.200507072

19. Montell DJ, Rorth P, Spradling AC. slow border cells, a locus required for a developmentally regulated cell migration during oogenesis, encodes Drosophila C/EBP. Cell. 1992;71: 51–62. doi:10.1016/0092-8674(92)90265-e

20. Sun J, Deng W-M. Hindsight mediates the role of notch in suppressing hedgehog signaling and cell proliferation. Dev Cell. 2007;12: 431–442. doi:10.1016/j.devcel.2007.02.003

21. Deady LD, Li W, Sun J. The zinc-finger transcription factor Hindsight regulates ovulation competency of Drosophila follicles. Elife. 2017;6. doi:10.7554/eLife.29887

22. Deady LD, Shen W, Mosure SA, Spradling AC, Sun J. Matrix Metalloproteinase 2 Is Required for Ovulation and Corpus Luteum Formation in Drosophila. PLOS Genetics. 2015;11: e1004989. doi:10.1371/journal.pgen.1004989

23. Ayme-Southgate A, Lasko P, French C, Pardue ML. Characterization of the gene for mp20: a Drosophila muscle protein that is not found in asynchronous oscillatory flight muscle. J Cell Biol. 1989;108: 521–531. doi:10.1083/jcb.108.2.521

24. Bai H, Kang P, Tatar M. Drosophila insulin-like peptide-6 (dilp6) expression from fat body extends lifespan and represses secretion of Drosophila insulin-like peptide-2 from the brain. Aging Cell. 2012;11: 978–985. doi:10.1111/acel.12000

25. Evans CJ, Liu T, Banerjee U. Drosophila hematopoiesis: markers and methods for molecular genetic analysis. Methods. 2014;68: 242–251. doi:10.1016/j.ymeth.2014.02.038
