# Supplementary material for: A single-cell atlas of adult Drosophila ovary identifies transcriptional programs and somatic cell lineage regulating oogenesis
Source: PLoS Biol. 2020 Apr 27;18(4):e3000538. doi: 10.1371/journal.pbio.3000538 (PMC7205450; doi:10.1371/journal.pbio.3000538)
Supplement: S4 File — Table of marker genes previously identified in different cell types with the associated references. Cells expressing 2 or more markers from different cell types were considered doublets and removed from the cluster (marked by X) to retain only high-quality cells. (PDF) [file pbio.3000538.s008.pdf]

<table>
<tr><th colspan="3" rowspan="2"></th><th colspan="5">Cluster identity for high-quality cell selection</th></tr>
<tr><th>Germline</th><th>Polar/Stalk/Mitotic FC</th><th>Mid-late FC</th><th>Muscle</th><th>Adipocyte</th></tr>
<tr><td rowspan="14">Cell-type markers used to select high-quality cells</td><td rowspan="3">Germline</td><td>osk [1–3]</td><td></td><td rowspan="2">X</td><td rowspan="2">X</td><td>X</td><td>X</td></tr>
<tr><td>bru-1 [4]</td><td></td><td></td><td></td></tr>
<tr><td>yl [4,5]</td><td></td><td></td><td></td><td rowspan="7">X</td><td rowspan="6">X</td></tr>
<tr><td rowspan="4">Mid-late FC</td><td>dec-1 [6,7]</td><td rowspan="7">X</td><td rowspan="4">X</td><td></td></tr>
<tr><td>psd [2,8,9]</td><td></td></tr>
<tr><td>Vm32E [6,10,11]</td><td></td></tr>
<tr><td>Vm26Ab [2,6,10]</td><td></td></tr>
<tr><td rowspan="3">Somatic</td><td>Vml [12]</td><td></td><td></td></tr>
<tr><td>tj [13]</td><td></td><td></td></tr>
<tr><td>Yp1/2/3 [6]</td><td></td><td></td><td></td></tr>
<tr><td rowspan="2">Muscle</td><td>Zasp66 [14]</td><td rowspan="2">X</td><td></td><td rowspan="2">X</td><td></td><td rowspan="2">X</td></tr>
<tr><td>Mp20 [15]</td><td></td><td></td></tr>
<tr><td>Adipocyte</td><td>Ilp6 [16,17]</td><td rowspan="2">X</td><td></td><td rowspan="2">X</td><td></td><td></td></tr>
<tr><td>Hemocyte</td><td>Hml [18]</td><td>X</td><td>X</td><td>X</td></tr>
</table>

References:

1. Sanghavi P, Liu G, Veeranan-Karmegam R, Navarro C, Gonsalvez GB. Multiple Roles for Egalitarian in Polarization of the Drosophila Egg Chamber. Genetics. 2016;203: 415–432. doi:10.1534/genetics.115.184622

2. Popodi E, Minoo P, Burke T, Waring GL. Organization and expression of a second chromosome follicle cell gene cluster in Drosophila. Dev Biol. 1988;127: 248–256. doi:10.1016/0012-1606(88)90312-0

3. Kim-Ha J, Smith JL, Macdonald PM. oskar mRNA is localized to the posterior pole of the Drosophila oocyte. Cell. 1991;66: 23–35. doi:10.1016/0092-8674(91)90136-M

4. Schonbaum CP, Lee S, Mahowald AP. The Drosophila yolkless gene encodes a vitellogenin receptor belonging to the low density lipoprotein receptor superfamily. PNAS. 1995;92: 1485–1489. doi:10.1073/pnas.92.5.1485

5. Schonbaum CP, Perrino JJ, Mahowald AP. Regulation of the vitellogenin receptor during Drosophila melanogaster oogenesis. Mol Biol Cell. 2000;11: 511–521. doi:10.1091/mbc.11.2.511

6. Tootle TL, Williams D, Hubb A, Frederick R, Spradling A. Drosophila Eggshell Production: Identification of New Genes and Coordination by Pxt. PLOS ONE. 2011;6: e19943. doi:10.1371/journal.pone.0019943

7. Noguerón MI, Mauzy-Melitz D, Waring GL. Drosophila dec-1 eggshell proteins are differentially distributed via a multistep extracellular processing and localization pathway. Dev Biol. 2000;225:459–470. doi:10.1006/dbio.2000.9805

8. Fakhouri M, Elalayli M, Sherling D, Hall JD, Miller E, Sun X, et al. Minor proteins and enzymes of the Drosophila eggshell matrix. Developmental Biology. 2006;293: 127–141. doi:10.1016/j.ydbio.2006.01.028

9. Elalayli M, Hall JD, Fakhouri M, Neiswender H, Ellison TT, Han Z, et al. Palisade is required in the Drosophila ovary for assembly and function of the protective vitelline membrane. Developmental Biology. 2008;319: 359–369. doi:10.1016/j.ydbio.2008.04.035

10. Bernardi F, Cavaliere V, Andrenacci D, Gargiulo G. Dpp signaling down-regulates the expression of VM32E eggshell gene during Drosophila oogenesis. Developmental Dynamics. 2006;235: 768–775. doi:10.1002/dvdy.20660

11. Gargiulo G, Gigliotti S, Malva C, Graziani F. Cellular specificity of expression and regulation of Drosophila vitelline membrane protein 32E gene in the follicular epithelium: identification of cis-acting elements. Mechanisms of Development. 1991;35: 193–203. doi:10.1016/0925-4773(91)90018-2

12. Zhang Z, Stevens LM, Stein D. Sulfation of Eggshell Components by Pipe Defines Dorsal-Ventral Polarity in the Drosophila Embryo. Current Biology. 2009;19: 1200–1205. doi:10.1016/j.cub.2009.05.050

13. Gunawan F, Arandjelovic M, Godt D. The Maf factor Traffic jam both enables and inhibits collective cell migration in Drosophila oogenesis. Development. 2013;140: 2808–2817. doi:10.1242/dev.089896

14. Hudson AM, Petrella LN, Tanaka AJ, Cooley L. Mononuclear muscle cells in Drosophila ovaries revealed by GFP protein traps. Dev Biol. 2008;314: 329–340. doi:10.1016/j.ydbio.2007.11.029

15. Ayme-Southgate A, Lasko P, French C, Pardue ML. Characterization of the gene for mp20: a Drosophila muscle protein that is not found in asynchronous oscillatory flight muscle. J Cell Biol. 1989;108: 521–531. doi:10.1083/jcb.108.2.521

16. Okamoto N, Yamanaka N, Yagi Y, Nishida Y, Kataoka H, O'Connor MB, et al. A Fat Body-Derived IGF-like Peptide Regulates Postfeeding Growth in Drosophila. Developmental Cell. 2009;17: 885–891. doi:10.1016/j.devcel.2009.10.008

17. Bai H, Kang P, Tatar M. Drosophila insulin-like peptide-6 (dilp6) expression from fat body extends lifespan and represses secretion of Drosophila insulin-like peptide-2 from the brain. Aging Cell. 2012;11: 978–985. doi:10.1111/acel.12000

18. Evans CJ, Liu T, Banerjee U. Drosophila hematopoiesis: markers and methods for molecular genetic analysis. Methods. 2014;68: 242–251. doi:10.1016/j.ymeth.2014.02.038
